# The ATP-independent dihydro-2-phenanthroyl-CoA reductase AprC catalyzes two consecutive two-electron reduction steps of dihydro-2-phenanthroyl-CoA to hexahydro-2-phenanthroyl-CoA in anaerobic phenanthrene degradation

**DOI:** 10.1128/aem.02248-25

**Published:** 2026-03-13

**Authors:** Nadia A. Samak, Khadija Adjir, Frederik Götz, Alina Surmeneva, Jonas Fax, Gebhard Haberhauer, Rainer U. Meckenstock

**Affiliations:** 1 Environmental Microbiology and Biotechnology (EMB), Faculty of Chemistry, University of Duisburg-Essen 27170, Essen, Germany; 2 Laboratory of Thermodynamics and Molecular Modeling, Faculty of Chemistry, University of Sciences and Technology Houari Boumediene (USTHB) 61758, Algiers, Algeria; 3 Organic Chemistry, Faculty of Chemistry, University of Duisburg-Essen 27170, Essen, Germany; Universidad de los Andes, Bogotá, Colombia

**Keywords:** old-yellow enzyme, NADH-flavin oxidoreductase, hydride transfer, PAHs, culture TRIP1, anaerobic phenanthrene degradation

## Abstract

Polycyclic aromatic hydrocarbons (PAHs) are widespread pollutants and especially difficult to degrade under anoxic conditions. The non-substituted three-ring PAH phenanthrene is activated through carboxylation to 2-phenanthroic acid which is then ligated to Co-enzyme A to produce 2-phenanthroyl-CoA, followed by step-wise reduction of the aromatic rings. The first reduction reaction is catalyzed by 2-phenanthroyl-CoA reductase producing dihydro-2-phenanthroyl-CoA. In this study, we elucidated the second reductase enzyme in the pathway, catalyzing the reduction of dihydro-2-phenanthroyl-CoA to hexahydro-2-phenanthroyl-CoA. Dihydro-2-phenanthroyl-CoA reductase (AprC) was heterologously overproduced in *Escherichia coli* and purified and characterized. AprC has a specific activity of 15.8 ± 0.3 nmol min^−1^ mg^−1^ and an apparent *K*_*m*_ value of 59.9 ± 3.8 nM at pH 7.5. Dithionite-reduced methyl viologen is the preferred *in vitro* electron donor for the four electron-reduction reaction, but it also functions with NADH but not with NADPH. The intermediate appearance of tetrahydro-2-phenanthroyl-CoA during the reduction to hexahydro-2-phenanthroyl-CoA indicates that AprC catalyzes two consecutive two-electron reduction steps. AprC is a monomeric enzyme with a molecular mass of ≈73 kDa and contains one FMN, one FAD, and one 4Fe-4S cluster, indicating that the reductase belongs to the type III aryl-CoA reductases of the old-yellow enzyme family. The high amino acid sequence similarity between dihydro-2-phenanthroyl-CoA reductase and 2-naphthoyl-CoA reductase, as well as the similar cofactor coordination in both enzymes, suggested that dihydro-2-phenanthroyl-CoA reductase follows the same biochemical principle as naphthalene ring reduction by 2-naphthoyl-CoA reductase. Quantum chemical calculation indicated that 5,6,7,8,9,10-hexahydro-2-phenanthroyl-CoA (isomer **6a**) is the most stable product of AprC out of eight possible isomers, although the real isomer is not known. The reductase also performed a two-electron reduction of chemically synthesized 9,10-dihydro-2-phenanthroyl-CoA to tetrahydro-2-phenanthroyl-CoA but at much lower reaction rates indicating that the 9,10-dihydro-2-phenanthroyl-CoA isomer is not the natural substrate of the enzyme and, thus, also not the real product of 2-phenanthroyl-CoA reduction by 2-phenanthroyl-CoA reductase AprB. Since 9,10-dihydro-2-phenanthroyl-CoA has two non-conjugated benzene rings, this also indicates that, surprisingly, type III aryl-CoA reductases can reduce benzene ring structures without additional ATP requirement.

**IMPORTANCE:**

Polycyclic aromatic hydrocarbons (PAHs) are highly toxic, persistent pollutants found in the environment. The anaerobic degradation of larger three-ring PAHs like phenanthrene is still poorly understood. Here, we show that after activation to a CoA-ester, the resonance energy of the aromatic ring system of phenanthrene is overcome by consecutive two-electron reduction steps catalyzed by ATP-independent type III aryl-CoA reductases belonging to the old-yellow enzyme family. This finding contributes to our understanding of the anaerobic degradation of PAHs with three or more rings.

## INTRODUCTION

The rapid growth of industrial activities has increased environmental pollution with polycyclic aromatic hydrocarbons (PAHs) which are highly carcinogenic (1, 2). The primary sources of PAHs in the environment include incomplete combustion of fossil fuels, biomass or waste, and direct spills of oil or PAHs into the environment, among others (3). PAHs spread in the environment and end up in soils and sediments, where they become recalcitrant to biodegradation due to the high chemical stability of the compounds and the low solubility in water (4). Furthermore, the high concentration of organic carbon in water-saturated sediments turns the environment anoxic due to the depletion of molecular oxygen rendering microbial degradation of PAHs very slow (5).

Anaerobic degradation of PAHs starts with activation of the compounds by fumarate addition for methylated PAHs or by carboxylation in the case of unsubstituted PAHs (6). The later activation reaction is followed by ligation with coenzyme A (CoA) to convert the carboxylic acids into thioesters (7, 8). The next step is the biochemically demanding reduction of the aromatic ring system which is needed to overcome the high resonance energy (9). There are three different strategies to reduce aromatic rings in anaerobic degradation. The first is catalyzed by ATP-dependent and oxygen-sensitive type I aryl-CoA reductases which utilize ferredoxin as a natural electron donor (10). The prototype is benzoyl-CoA reductase which is commonly found in benzoyl-CoA **[13]** degradation by facultative anaerobes such as *Thauera aromatica* (11) or *Rhodopseudomonas palustris* (12). The second strategy comprises ATP-independent and oxygen-sensitive type II aryl-CoA reductases which also reduce benzoyl-CoA. These enzymes contain tungsten (11) and were discovered in strict anaerobes such as the iron reducer *Geobacter metallireducens* and the sulfate reducer *Desulfococcus multivorans* (13–18). The third type is the ATP-independent and oxygen-insensitive type III aryl-CoA reductases which belong to the old-yellow enzyme family (OYE) and are only known for anaerobic degradation of PAHs, so far (9, 19–21). In the anaerobic degradation of naphthalene **[7]**, the aromatic ring system of the key metabolite 2-naphthoyl-CoA **[9]** is first reduced by the ATP-independent type III 2-naphthoyl-CoA-reductase to 5,6-dihydro-2-naphthoyl-CoA **[10]**. Then follows another reduction by a second type III reductase, 5,6-dihydro-2-naphthoyl-CoA-reductase (21). The resulting 5,6,7,8-tetrahydro-2-naphthoyl-CoA **[11]** is then reduced by an ATP-dependent type I aryl-CoA reductase to hexahydro-2-naphthoyl-CoA **[12]** which can be further metabolized by beta-oxidation like reactions (19, 20).

**Fig 1:**
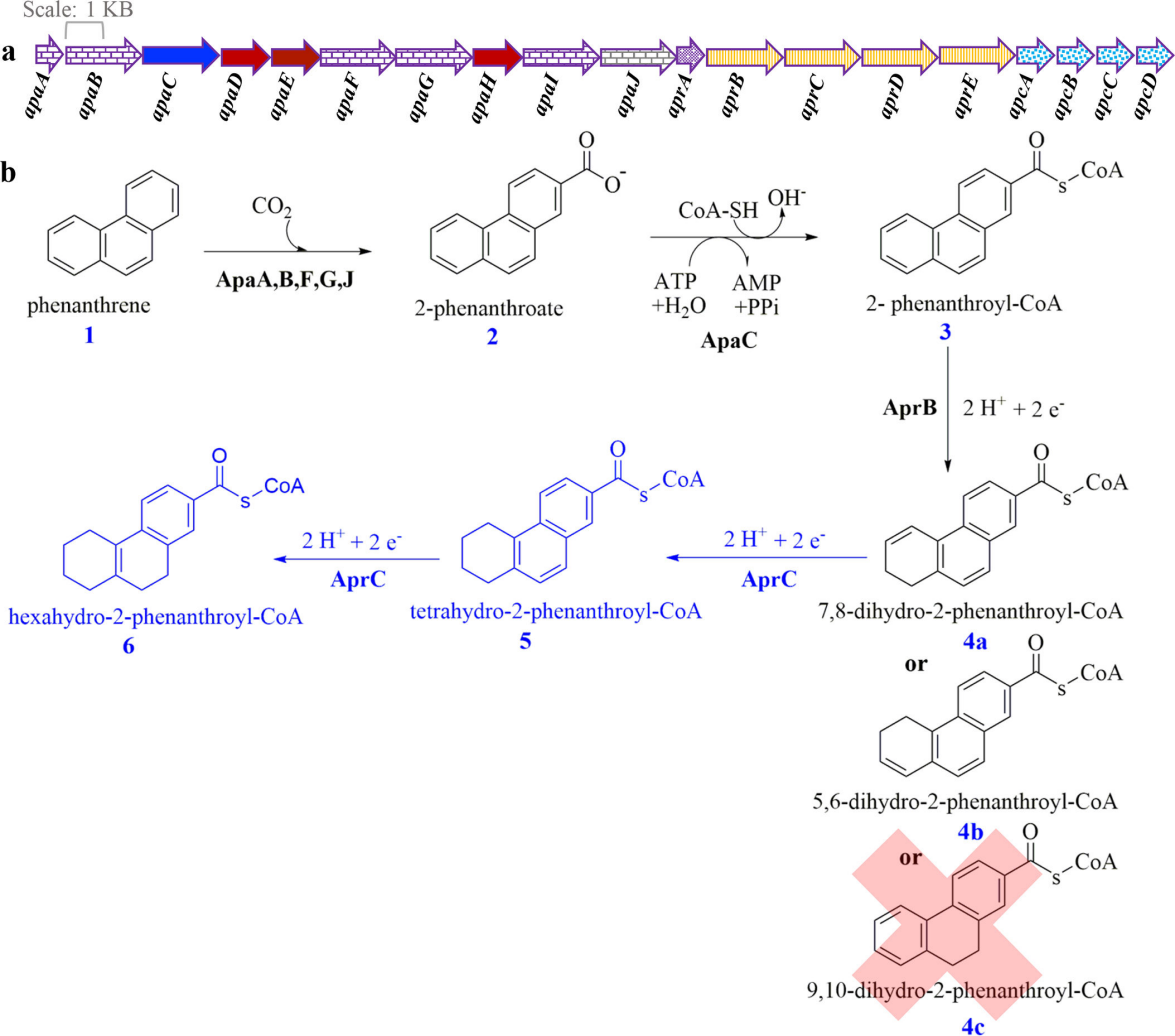
Schematic drawing of the gene cluster encoding the upper degradation pathway of anaerobic phenanthrene degradation (**a**) and the proposed degradation pathway (**b**). (**a**) Genes depicted in purple horizontal brick (*apaA,B,F,G, I*, and *J*) are proposed to encode the phenanthrene carboxylase subunits because of their similarity to the UbiD-like enzyme naphthalene carboxylase (22). The gene depicted in solid blue (*apaC*) encodes 2-phenanthroate:CoA ligase investigated in a former study (5). Genes depicted in solid red (*apaD, E*, and *H*) are similar to genes for ParA/MinD ATPase like, MRP, Fer4_NifH superfamily and conserved hypothetical proteins with unknown function. The gene depicted in dotted purple (*aprA*) is proposed to encode a ferredoxin, providing low-potential electrons for aryl-CoA reductases, needed for anaerobic degradation of PAHs (24). Genes depicted in yellow vertical strips (*aprB-E*) encode 2-phenanthroyl-CoA reductase (23), dihydro-2-phenanthroyl-CoA reductase (investigated in this study), a putative hexahydro-2-phenanthroyl-CoA reductase, and a putative octahydro-2-phenanthroyl-CoA reductase, respectively (9, 25). Genes depicted in light blue large confetti are proposed to encode the β-oxidation-like enzymes that could be responsible for opening the reduced phenanthroyl-CoA ring system as deduced from the similarity to enzymes in the naphthalene degradation pathway (22). (**b**) Proposed partial anaerobic phenanthrene degradation pathway. Verified enzyme reactions are illustrated in black, while the enzyme reactions investigated in this study are highlighted in blue. The indicated isomers of dihydro-2-phenanthroyl-CoA **[4]** and 5,6,7,8,9,10-hexahydro-2-phenanthroyl-CoA **[6]** are only proposed structures since the exact positions of the saturated bonds are not known yet.

The anaerobic biodegradation of small PAHs such as naphthalene **[7]** is at least partially studied (19), but the anaerobic biodegradation of larger PAHs such as phenanthrene **[1]** is much less understood. We enriched the culture TRIP 1 from oil-contaminated soil near the Pitch Lake in Trinidad, which can anaerobically oxidize phenanthrene **[1]** as the sole electron source to CO_2_ with sulfate as electron acceptor (1). So far, phenanthrene **[1]** follows the same degradation mechanism as naphthalene **[7]**, starting from phenanthrene **[1]** activation through carboxylation to phenanthroic acid **[2]** and CoA-ligation to phenanthroyl-CoA **[3]** (Fig. 1) (1, 5, 22). A cluster of genes in the genome of candidate strain TRIP 1 encodes for enzymes involved in anaerobic phenanthrene degradation (22, 23). Genes *apaA–apaJ* encode the phenanthrene carboxylase subunits including the *apaC* which encodes 2-phenanthroate:CoA ligase enzyme (Fig. 1a). Four genes encoding NADH-flavin oxidoreductases belonging to the old-yellow enzyme family are located in the genome of the sulfate-reducing enrichment culture TRIP1 (*aprB-E*, Fig. 1a) (23). The first gene encodes 2-phenanthroyl-CoA reductase (AprB), which belongs to type III aryl-CoA reductase, and reduces one double bond in ring 3 of 2-phenanthroyl-CoA **[3]** producing dihydro-2-phenanthroyl-CoA **[4a** or **4b]** to overcome the resonance energy of the aromatic ring (23) similar to naphthoyl-CoA reductase (21).

In this study, we investigated the second ring reduction reaction converting dihydro-2-phenanthroyl-CoA **[4a, 4b,** or **4c]** to hexahydro-2-phenanthroyl-CoA **[6]**. We heterologously expressed the dihydro-2-phenanthroyl-CoA reductase encoding gene (*aprC*) in *E. coli* Bl21(DE3) and purified and characterized the produced enzyme.

## MATERIALS AND METHODS

### Cloning of dihydro-2-phenanthroyl-CoA reductase encoding gene (*aprC*)

The NADH-flavin oxidoreductase *aprC* gene sequence from TRIP 1 (PITCH_A1860005) (22) was codon optimized for expression in *Escherichia coli* at Eurofins Genomics, Ebersberg, Germany. Both sequences of the original and codon-optimized *aprC* gene are provided in the supplementary materials (Fig. S1). The codon-optimized *aprC* gene was amplified by PCR using a 2× KAPA HiFi HotStart ready mix (Fisher Scientific GmbH, Schwerte, Germany) and the forward primer AGCGCGTCTCCAATGTCCAATCGCTTTGTGAACC and the reverse primer AGCGCGTCTCCTCCCCCAGCTAACCAACCTTCGTG. PCR products were purified using the Monarch PCR and DNA Cleanup kit (New England Biolabs GmbH, Frankfurt, Germany). The purified amplicon (2 nM) was cut with the restriction enzymes Esp3I (2.5 µL), and Esp3I (5 U) and the gene was integrated into the pASG-IBA103 expression plasmid (5 ng) using 250 mM DTT/12.5 mM ATP mix (1 µL), T4 DNA ligase (1 U), followed by incubation for 1 h at 30°C. The recombinant pASG-IBA103-*aprC* was first transformed into NEB-5α competent *E. coli* cells and then into BL21(DE3) *E. coli* cells for heterologous overproduction. The recombinant plasmid was extracted and sequenced to confirm successful cloning (Eurofins Genomics, Ebersberg, Germany).

### Overproduction and purification of dihydro-2-phenanthroyl-CoA reductase

The expression of dihydro-2-phenanthroyl-CoA reductase encoding gene (*aprC*) was performed by growing *E. coli* BL21(DE3) carrying the recombinant pASG-IBA103-*aprC* in 1L flask containing 500 mL Lysogeny broth (LB) supplemented with 100 µg/mL ampicillin and riboflavin (≈0.3 g) at 37°C with shaking (200 rpm) until the optical density (OD600) reached 0.5–0.6. The expression of the gene encoding dihydro-2-phenanthroyl-CoA reductase (*aprC*) was stimulated with 0.2 µg/mL anhydrotetracycline. Then, the culture was incubated at 20°C for 20 h at 130 rpm. The cells were collected using centrifugation (3,100 × *g*, 30 min), and the pellet was resuspended in 50 mM HEPES buffer (pH 8, 2 mL buffer per g wet weight *E. coli* cells, 10 g wet weight *E. coli* cells were produced per Liter culture medium). Cell disruption was carried out with a French pressure cell (83 bar), and the cell debris was removed by 1 h of centrifugation (16,000 × *g*) in 2 mL reaction tubes at 4°C. The supernatant (20 mL) containing the overproduced dihydro-2-phenanthroyl-CoA reductase was loaded onto gravity flow Strep-Tactin XT 4Flow columns (IBA, Göttingen, Germany) and eluted with 50 mM biotin dissolved in 50 mM HEPES buffer containing 150 mM NaCl, in three fractions with 3, 8, and 4 mL, respectively. Purified protein was concentrated using a Pierce Protein Concentrator (ThermoFisher Scientific) with a cut-off of 50 kDa. The protein concentration was evaluated using the Bradford test (26), and purity was assessed using SDS-PAGE with a 12% (wt/vol) Mini-PROTEAN TGX gel (BioRad, USA). The molecular mass of the pure enzyme was detected using ThermoScientific’s PageRuler Prestained Protein Ladder.

### Gene abbreviations involved in phenanthrene degradation

The abbreviation concept for genes mentioned in this study is as follows: apa, for anaerobic phenanthrene activation; apr, anaerobic phenanthroyl-CoA reduction; apc, anaerobic phenanthrene ring cleavage. Compounds mentioned in this manuscript were numbered between square brackets in the whole text and colored in blue in all figures.

### Synthesis of 2-phenanthroyl-CoA

2-Phenanthroyl-CoA ester **[3]** was synthesized by mixing 4.2 mg of carbonyldiimidazole with 32 µM of 2-phenanthroic acid **[2]** in tetrahydrofuran (THF, 200 µL) and incubating at 900 rpm and 22°C in a thermomixer (Eppendorf, Germany) in an anaerobic chamber for 2 h. Then, 5 mg of CoA was dissolved in 250 µL sodium bicarbonate (100 mM) and added to the THF solution and incubated for 5 h. Ten microliters of aqueous formic acid (20%, v/v) was added to the previous mixture, and 100 µL ethyl acetate was used to remove all apolar compounds as well as the THF. The organic phase was discarded, and the aqueous phase containing the 2-phenanthroyl-CoA ester **[3]** was stored at −70°C and then freeze-dried using a lyophilizer (Martin Christ Gefriertrocknungsanlagen GmbH, Osterode am Harz, Germany) overnight. The dry 2-phenanthroyl-CoA ester **[3]** (≈2–2.5 mg) was dissolved in 2 mL of 0.1% (vol/vol) formic acid and purified with SPE columns (CHROMABOND C18 Hydra, 45 µm, 6 mL, MACHEREY-NAGEL GmbH, Dueren, Germany). The column was activated with acetonitrile (2 mL) and equilibrated with the same amount of 0.1% (vol/vol) formic acid. The dissolved 2-phenanthroyl-CoA ester **[3]** was loaded on the column and washed twice with 2 mL of 0.1% (vol/vol) formic acid. The CoA-ester was purified by serial elution of acetonitrile (5%, 10%, 20%, 40%, 60%, and 100%) in 0.1% (vol/vol) aqueous formic acid (each fraction 4 mL). The successful synthesis of 2-phenanthroyl-CoA ester **[3]** was checked with LC-2040C system coupled to a LC-MS-2020 single quadrupole mass-spectrometer (Shimadzu Deutschland, Duisburg, Germany), and the fraction with the highest concentration of the purified substrate was freeze-dried and the powder stored at −20°C.

### Synthesis and analysis of dihydro-2-phenanthroyl-CoA

Since the substrate dihydro-2-phenanthroyl-CoA **[4]** is not commercially available, the compound was produced enzymatically by reducing chemically synthesized 2-phenanthroyl-CoA **[3]** with 2-phenanthroyl-CoA reductase (AprB), encoded by the gene PITCH_a10001 (*aprB*), which was described previously (23).

Dry 2-phenanthroyl-CoA **[3]** was taken up in 50 mM HEPES buffer, pH 7.5 (100 µM). The reduction assay was performed in 200 µL volume containing 100 µL of the concentrated, purified phenanthroyl-CoA reductase (AprB) (100 µg) and 100 µL of a mixture of 1 mM NADH, 50 µM FMN, 1 mM FAD, 1 mM methyl viologen, and 1 mM dithionite dissolved in 50 mM HEPES buffer, pH 7.5. The enzymatic reduction assay was incubated for 90 min, and the reaction was stopped by adding a double volume of methanol. The methanol was evaporated using a speedDry Vacuum Concentrator (Martin Christ Gefriertrocknungsanlagen GmbH, Osterode am Harz, Germany), and the aqueous solution containing the enzymatically reduced dihydro-2-phenanthroyl-CoA **[4a** or **4b]** was freeze-dried overnight and then used as substrate.

### Determination of dihydro-2-phenanthroyl-CoA reductase activity

The reduction assay (200 µL) contained 100 µL of a mixture of 1 mM NADH, 50 µM FMN, 1 mM FAD, and 30 µM dihydro-2-phenanthroyl-CoA ester **[4a** or **4b]**, dissolved in 50 mM HEPES buffer, pH 7.5. The concentrated, purified dihydro-2-phenanthroyl-CoA reductase (AprC) (50-100 µg dissolved in 100 µL of 50 mM HEPES buffer, pH 7.5) was added to the reaction mixture to initiate the reaction, which was conducted at 30°C with shaking at 900 rpm in a Thermomix Block (ThermoMixer C, Eppendorf, Germany) within an anaerobic chamber with nitrogen atmosphere (O_2_ < 0.5 ppm) (M. Braun Inertgas-Systeme GmbH, Garching, Germany). After 0, 45, and 90 min, 40 µL aliquots were taken into an Eppendorf tube, and the reaction was stopped by mixing with a double volume of methanol. The tubes were centrifuged (16,000 × *g*) for 60 min to remove precipitates of protein and salts, and the supernatants were transferred to LC-MS vials to analyze the reduction products, hexahydro-2-phenanthroyl-CoA **[6]**.

Potential electron donors were tested and added to the assays in final concentrations: sodium dithionite (5 mM), Ti(III)-citrate (5 mM), NADPH (5 mM), and dithionite (1 mM) reduced methyl viologen (1 mM). The reaction was also carried out in the presence and absence of 50 µM ATP to determine potential ATP-dependence.

### LC-MS analysis

The analyses were conducted with an LC-2040C system connected to a single quadrupole mass spectrometer (LC-MS-2020) (Shimadzu, Duisburg, Germany) as described before (23). Briefly, the metabolites were separated using a Nucleodur C18 Gravity-SB column (250 mm column length, 4.6 mm column inner diameter, 5 μm particle size, Macherey-Nagel GmbH, Düren, Germany) at a temperature of 35°C. Compounds were developed with a linear gradient starting with 90% solvent A (0.1% [wt/vol] ammonium formate) and 10% solvent B (acetonitrile) and transitioning to 90% solvent B over 30 min, with a flow rate of 0.4 mL/min. The analytes were detected with an electrospray ionization (ESI) system in both positive and negative ionization modes. The ion spray voltage was set at 4,500 V for positive mode and −4,500 V for negative mode, at a temperature of 350°C. Besides targeting specific molecular masses of potential metabolites, mass-to-charge ratios ranging from 50 to 1,050 *m*/*z* were also scanned.

### UV-vis spectroscopy

The reduction product, hexahydro-2-phenanthroyl-CoA **[6]**, was also monitored using an Infinite M200 Pro TECAN Spectrophotometer (Tecan Group Ltd., Switzerland). Furthermore, UV/vis analysis was performed for standard solutions of coenzyme A, 2-phenanthroyl-CoA **[3]**, and 9,10-dihydro-2-phenanthroyl-CoA **[4c]**.

### Dihydro-2-phenanthroyl-CoA reductase kinetic properties

Dihydro-2-phenanthroyl-CoA reductase specific activity was measured based on the conversion of the substrate to hexahydro-2-phenanthroyl-CoA **[6]** within the first 10 min. Specific activities were calculated from an average of three independent replicates. The enzyme assay of dihydro-2-phenanthroyl-CoA reductase was conducted using various initial concentrations of dihydro-2-phenanthroyl-CoA **[4a** or **4b]** (ranging from 1 to 300 μM) in order to establish the initial reaction rate, the apparent Michaelis constant (*K_m_*), and the maximum reaction rate (*V*_*max*_).

### Determination of flavin and iron content in dihydro-2-phenanthroyl-CoA reductase

The presence of flavins in dihydro-2-phenanthroyl-CoA reductase was analyzed by UV/vis absorption spectroscopy with an Infinite M200 Pro TECAN Spectrophotometer (Tecan Group Ltd., Switzerland). The purified enzyme (20 µM) was put into nitrogen-flushed, stoppered quartz cuvettes and gradually reduced by step-wise addition of sodium dithionite (0.05 mM). The flavin content of dihydro-2-phenanthroyl-CoA reductase was analyzed with extraction and LC-MS as described earlier (9, 23). The iron content was determined using an iron ferrozine colorimetric method adapted from (27).

### Sequence alignment

The protein sequences of the genes PITCH_A1860005 (*aprC*), PITCH_a10001 (*aprB*), and N47_G38220 encoding dihydro-2-phenanthroyl-CoA reductase, 2-phenanthroyl-CoA reductase, and 2-naphthoyl-CoA reductase, respectively, were obtained from the *UniProt* database (28). The alignment was performed with the Biotite Python package. A BLOSUM62 matrix with gap penalties of −10 and −1, and without terminal gap penalty, was used for the alignment (29).

### Complex modeling and visualization

The predicted structures of 2-phenanthroyl-CoA reductase (AprB) and dihydro-2-phenanthroyl-CoA reductase (AprC) were downloaded from the AlphaFold Protein Structure Database (30). To populate the structures with the cofactors FMN, FAD, and the 4Fe-4S cluster, *AlphaFill* was used with its *YASARA* optimization function to transfer the cofactors from the 2-naphthoyl-CoA reductase protein structure (6QKG) to AprB and from 2,4-dienoyl-CoA reductase (1PS9) to AprC (31). To compare the structure of the protein, they were aligned using PyMol. The inner cavities were visualized using CavitOmiX (v. 1.0, 2022, Innophore GmbH). VASCo was used to analyze the hydrophobicity of the pockets (32–34). Cavities were calculated using a modified LIGSITE algorithm (35).

### Quantum chemical calculations

Eight putative isomers of the reaction product hexahydro-2-phenanthroyl-CoA **[6]**, produced from the reduction of the enzymatically accumulated dihydro-2-phenanthroyl-CoA **[4a** or **4b]**, and five putative isomers of the product tetrahydro-2-phenanthroyl-CoA **[5]** produced from the reduction of chemically synthesized 9,10-dihydro-2-phenanthroyl-CoA **[4c]** were selected based on their energy stabilities. 9,10-Dihydro-2-phenanthroyl-CoA **[4c]** was chemically synthesized as described previously (23). The relative energies of isomers of the reduction products hexahydro-2-naphthoyl-CoA **[12]** and cyclohexa-1,5-diene-1-carbonyl-CoA **[13]**, which are metabolites in anaerobic naphthalene degradation, were also calculated for comparison. To investigate the relative stability of all metabolites, geometry optimizations were performed using Density Functional Theory (DFT) (36), at the B3LYP level with the 6-311+G(d,p) basis set (37, 38), via the Gaussian 16 program package (39). The Polarizable Continuum Model (PCM) was employed to incorporate solvent effects, with methanol as the solvent medium (40–42). All optimized structures exhibited C₁ symmetry, indicating no molecular symmetry constraints. Harmonic vibrational frequency calculations were carried out for each structure to verify that they correspond to true minima on the potential energy surface, as evidenced by the absence of imaginary frequencies. The electronic energy difference was used to determine the relative stability of the reduction products. It represents the difference in total electronic energies between the products and reactants. The electronic energy itself is the total energy of the electrons in a molecule, determined by their interactions with the nuclei and with each other. It reflects the intrinsic, quantum-mechanical energy of a molecule and is often used to compare the relative stability of chemical species (43).

### Reduction reaction


$$\Delta E_{elec}=\sum \left(E_{products}\right)-\sum \left(E_{reactants}\right)$$

The enthalpy changes Δ*H*₍₂₉₈₎, which includes the electronic energy together with vibrational, rotational, translational, and PV (pressure–volume) contributions (ZPE and thermal corrections), was also calculated. It represents the energy absorbed or released at constant pressure and was determined to describe energy changes at real temperature (298 K). In addition, the Gibbs free energy ΔG₍₂₉₈₎, which includes both enthalpy and entropy terms, was determined to evaluate the spontaneity of the reduction reaction (43).

Thus:


$$\;\;\;\;\;\;\;\;\;\;\;\Delta H_{\left(298\right)}\approx \Delta E_{elec}+\Delta E_{ZPE}+\Delta H_{therm}$$

$$\;\;\;\;\;\;\;\;\;\;\;\Delta G_{\left(298\right)}=\Delta H_{\left(298\right)}-T\Delta S_{\left(298\right)}$$

When:

Δ*E*_elec_ is the electronic energy difference, Δ*E*_ZPE_ is the Zero-point energy correction, Δ*H*_therm_ is the thermal enthalpy correction, and Δ*S* is the entropy term for Gibbs free energy.

Δ*E*_ZPE_ = Σ(ZPE_products_) − Σ(ZPE_reactants_)

Δ*H*_therm_ = Σ*H*_corr_products_ − Σ*H*_corr_reactants_

Δ*S*₍₂₉₈₎ = Σ*S*_products_ − Σ*S*_reactants_

## RESULTS AND DISCUSSION

### Biochemical characterization of the overproduced and purified dihydro-2-phenanthroyl-CoA reductase

**Fig 2:**
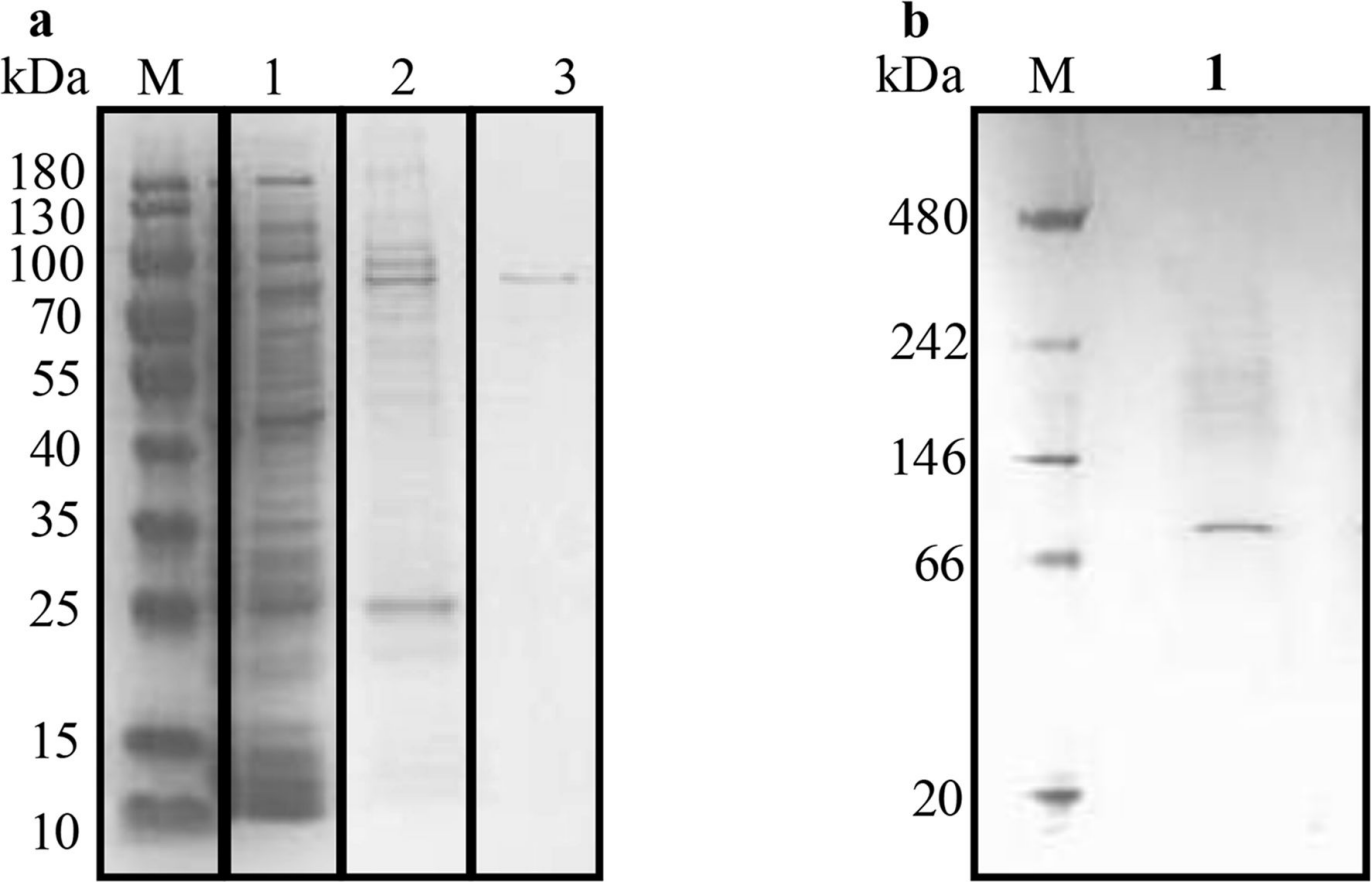
(**a**) SDS-PAGE of fractions from the purification procedure of dihydro-2-phenanthroyl-CoA reductase. M, protein MW standard; lane 1, *E. coli* cell-free extract; lanes 2 and 3 show two different elution fractions of the Strep-Tactin affinity-column with biotin (50 mM) buffer as eluent. (**b**) Blue native-PAGE analysis of the heterologously overproduced and purified dihydro-2-phenanthroyl-CoA reductase (lane 1).

The *aprC* gene was cloned into the expression plasmid PASG-IBA103 for the heterologous overproduction of a C-terminal Twin-Strep-tag-fusion protein. The reductase enzyme AprC was aerobically overproduced in *E. coli* BL21(DE3) resulting in the production of soluble dihydro-2-phenanthroyl-CoA reductase AprC (≈3 mg of protein was produced from an *E. coli* wet mass of 10 g). The produced AprC reductase was purified using Strep-Tactin XT 4Flow gravity flow column, and the enzyme purification was confirmed by SDS-PAGE analysis, which showed pure enzyme with a molecular mass of ≈73 kDa (Fig. 2a). The native state of the purified enzyme was confirmed by blue native gel electrophoresis, which showed a monomeric enzyme with a molecular mass of ≈73 kDa (Fig. 2b).

**Fig 3:**
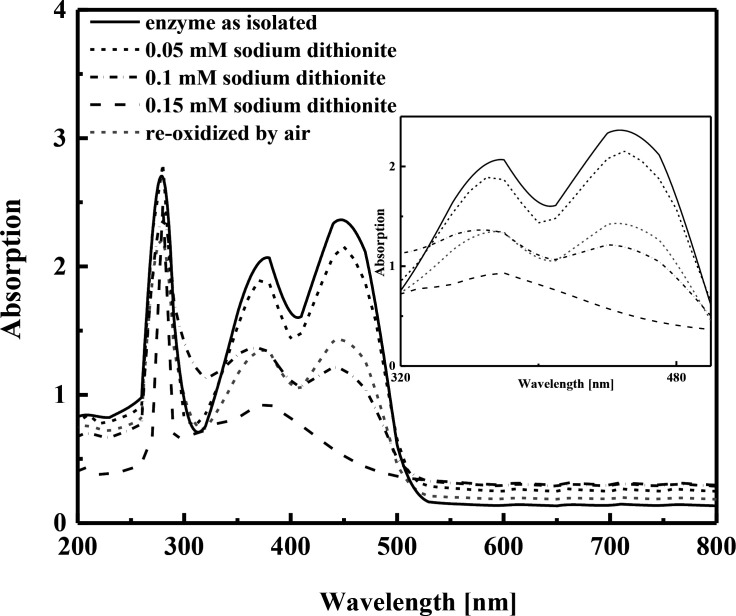
UV/vis spectra of dihydro-2-phenanthroyl-CoA reductase (20 µM) as isolated and after sequential addition of sodium dithionite (0.05 mM steps) as well as re-oxidized by exposure to air. The absorbance maximum at approximately 280 nm corresponds to the aromatic amino acid residues of AprC.

The gene sequence of the dihydro-2-phenanthroyl-CoA reductase suggested that it belongs to the old yellow enzyme family, which was supported by its yellow color indicating the presence of flavin cofactors. The UV/vis spectrum of dihydro-2-phenanthroyl-CoA reductase as isolated showed two distinct absorption maxima at 375 and 450 nm which are indicative of oxidized FMN and FAD (Fig. 3). The intensity of the two peaks progressively decreased upon gradual addition of 0.05 mM sodium dithionite until complete reduction was achieved with 0.15 mM sodium dithionite. The re-oxidation of the reduced enzyme was simply accomplished by exposing the solution to air, which restored the characteristic flavin peaks in the UV-vis spectrum (Fig. 3). The flavin content was analyzed with LC/MS and commercial standards, yielding an average of 0.7 ± 0.1 FMN per monomer and 0.8 ± 0.4 FAD per monomer (Table 1), suggesting that each monomer contained one FMN and one FAD. To investigate the presence of 4Fe-4S clusters in the enzyme, the iron content was measured colorimetrically, resulting in an average value of 3.3 ± 0.5 Fe per monomer, suggesting the presence of one 4Fe-4S cluster per monomer (Table 1). The presence of one FMN, FAD, and a 4Fe-4S cluster per enzyme monomer suggests that the enzyme belongs to the ATP-independent type III aryl-CoA reductases of the old-yellow enzyme family.

**TABLE 1:** Biochemical characteristics of dihydro-2-phenanthroyl-CoA reductase

Parameter	Value
Specific activity at *V*_max_	15.8 ± 0.3 nmol min^−1^ mg^−1^
Apparent *K*_m_	59.9 ± 3.8 nM
FMN content	0.7 ± 0.1 FMN/monomer
FAD content	0.8 ± 0.4 FAD/monomer
Iron content	3.3 ± 0.5 Fe/monomer
Used electron donors (activity of other electron donors given in % of the maximal activity measured with reduced methyl viologen)	Dithionite-reduced methyl viologen: 100% Dithionite: 35% NADH: 40% Ti(III)-citrate: 0% NADPH: 0%
Enzymatic assay oxygen sensitivity	Yes

### Dihydro-2-phenanthroyl-CoA reductase catalysis two consecutive two-electron reduction steps of dihydro-2-phenanthroyl-CoA

In the anaerobic degradation of phenanthrene **[1]**, the aromatic hydrocarbon is first activated through carboxylation to produce 2-phenanthroic acid **[2]** which is catalyzed by phenanthrene carboxylase (Fig. 1). Phenanthroic acid **[2]** is then transformed by 2-phenanthroate:CoA ligase (ApaC) to produce 2-phenanthroyl-CoA **[3]** (5, 19). The degradation continues through a two-electron reduction of 2-phenanthroyl-CoA **[3]** catalyzed by 2-phenanthroyl-CoA reductase (AprB) to produce dihydro-2-phenanthroyl-CoA with two possible isomers 7,8-dihydro-2-phenanthroyl-CoA **[4a]** [IUPAC: 1,2-dihydro-7-phenanthroyl-CoA] or 5,6-dihydro-2-phenanthroyl-CoA **[4b]** [IUPAC: 3,4-dihydro-7-phenanthroyl-CoA] (23). Unfortunately, the reduced product was not stable in our former study and tended to undergo isomerization changes to reach the more stable 9,10-dihydro-2-phenanthroyl-CoA **[4c]** (Fig. 1) which was identified with nuclear magnetic resonance spectroscopy (NMR) (23). However, the chemically synthesized reference compound 9,10-dihydro-2-phenanthroyl-CoA **[4c]** was only converted in minimal amounts by dihydro-2-phenanthroyl-CoA reductase (AprC) compared to the real metabolite 5,6-dihydro-2-phenanthroyl-CoA **[4b]** or 7,8-dihydro-2-phenanthroyl-CoA **[4a]**, enzymatically produced by using 2-phenanthroyl-CoA reductase (AprB). This indicates that one of the two later isomers is the natural substrate for the second reduction reaction by dihydro-2-phenanthroyl-CoA reductase (AprC) rather than the thermodynamically more stable compound 9,10-dihydro-2-phenanthroyl-CoA **[4c]**.

**Fig 4:**
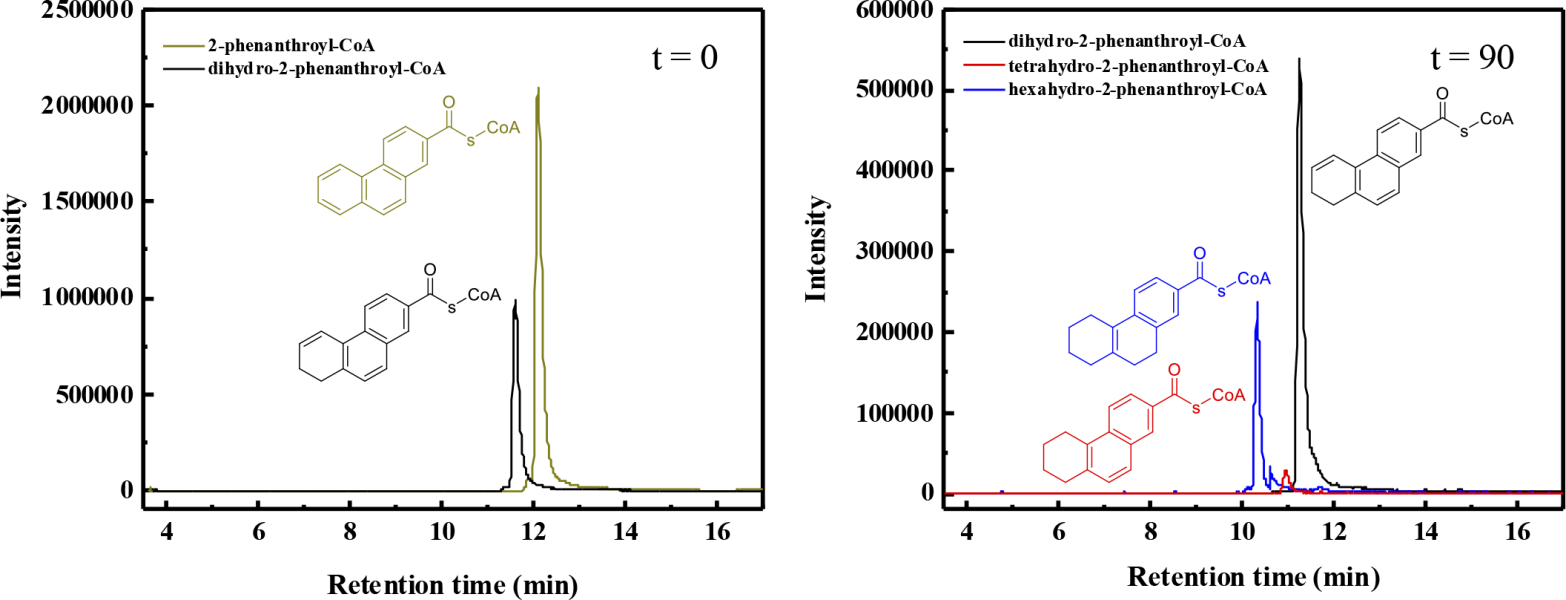
LC-MS analysis showing the *in vitro* conversion of the enzymatically accumulated dihydro-2-phenanthroyl-CoA **[4a** or **4b]** (substrate) to the reduced product, hexahydro-2-phenanthroyl-CoA **[6]**. The graph at 0 min shows the result of the first reduction reaction to convert the chemically synthesized 2-phenanthroyl-CoA **[3]** enzymatically to dihydro-2-phenanthroyl-CoA **[4a** or **4b]**, which is used as substrate for dihydro-2-phenanthroyl-CoA reductase. The result of the following four-electron reduction reaction employing dihydro-2-phenanthroyl-CoA reductase, AprC, is shown at 90 min. The black line indicates the leftover of the enzymatically accumulated substrate, dihydro-2-phenanthroyl-CoA **[4a** or **4b]** (*m*/*z* = 974, positive ion mode). The blue line depicts the produced hexahydro-2-phenanthroyl-CoA **[6]** after 90 min (*m*/*z* = 978, positive ion mode). The red line depicts the produced tetrahydro-2-phenanthroyl-CoA **[5]** (*m*/*z* = 976, positive ion mode).

In this study, we revealed the next reduction step, which converts the dihydro-2-phenanthroyl-CoA **[4a, 4b,** or **4c]** to tetrahydro- **[5]** and hexahydro-2-phenanthroyl-CoA **[6]** by dihydro-2-phenanthroyl-CoA reductase (AprC). One of the challenges faced in conducting the reduction experiments of the dihydro-2-phenanthroyl-CoA **[4a, 4b,** or **4c]** to hexahydro-2-phenanthroyl-CoA **[6]** was that the substrates for the enzyme reactions are not commercially available. Hence, the substrate dihydro-2-phenanthroyl-CoA **[4a or 4b]** was produced enzymatically by reducing chemically synthesized 2-phenanthroyl-CoA **[3]** with heterologously overproduced 2-phenanthroyl-CoA reductase (AprB) as described before (23) in order to accumulate larger amounts of dihydro-2-phenanthroyl-CoA **[4a** or **4b]** in the assay. Then, the dihydro-2-phenanthroyl-CoA reductase was added to the enzymatic assay containing the accumulated dihydro-2-phenanthroyl-CoA **[4a** or **4b]** as substrate. The reduction reaction needed dithionite-reduced methyl viologen as electron donor. To obtain better activity, 1 mM NADH, 50 µM FMN, and 1 mM FAD were added to the enzyme assay. The catalytic activity of the enzyme was significantly less with either dithionite or NADH only as electron donors (35% and 40% of the dithionite-reduced methyl viologen). There was no catalytic activity with Ti(III)-citrate or NADPH as electron donor (Table 1). The dihydro-2-phenanthroyl-CoA reductase catalyzed a four-electron reduction producing hexahydro-2-phenanthroyl-CoA [6] with a specific activity of 15.8 ± 0.3 nmol min^−1^ mg^−1^ (Fig. 4), which was slightly lower compared to the specific activity of 2-phenanthroyl-CoA reductase (17.6 ± 0.4 nmol min^−1^ mg^−1^), which converts 2-phenanthroyl-CoA **[3]** to 5,6-dihydro-2-phenanthroyl-CoA **[4b]** or 7,8-dihydro-2-phenanthroyl-CoA **[4a]** (23). There were also small amounts of tetrahydro-2-phenanthroyl-CoA **[5]** detectable indicating the overall reaction comprises two consecutive two-electron reduction steps (Fig. 4).

The initial rate of dihydro-2-phenanthroyl-CoA **[4a** or **4b]** reduction to hexahydro-2-phenanthroyl-CoA **[6]** was examined from three independent experiments using various initial concentrations of dihydro-2-phenanthroyl-CoA **[4a** or **4b]** ranging from 1 to 300 μM, which followed Michaelis-Menten kinetics with an apparent *K*_*m*_ value of 59.9 ± 3.8 nM (mean value ± SD, Table 1). The four-electron reduction reaction was ATP-independent confirming that dihydro-2-phenanthroyl-CoA reductase belongs to the ATP-independent class III of aryl-CoA reductases. The addition of ATP did not affect the reaction rate. The enzymatic reduction assay with AprC could only be performed in the absence of oxygen to prevent the chemical oxidation of dithionite-reduced methyl viologen used as an electron donor. The enzyme itself, however, is not oxygen sensitive because it was purified under ambient air and was still active in the enzyme assays.

**Fig 5:**
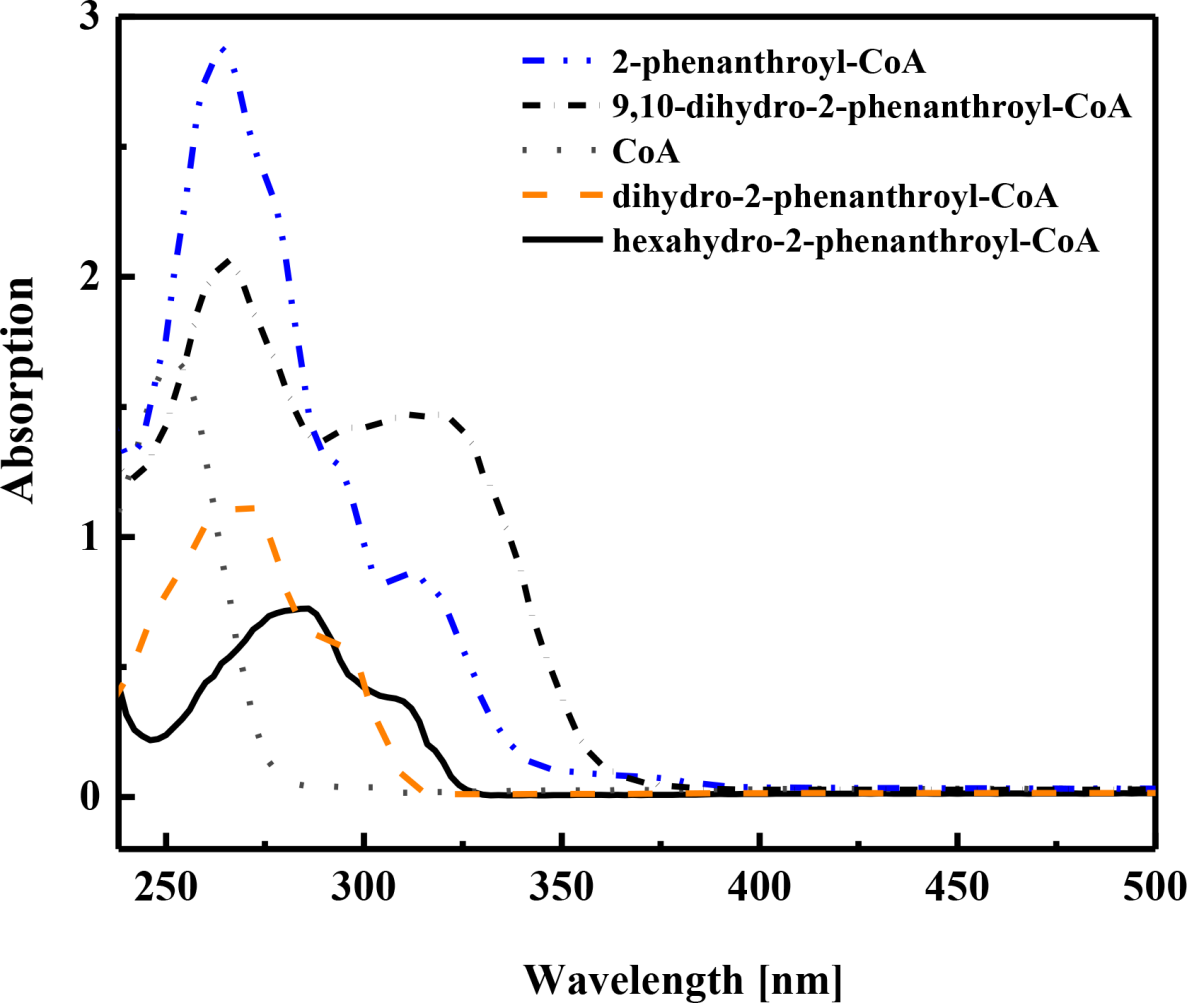
UV/vis absorption spectra of the starting substrate dihydro-2-phenanthroyl-CoA **[4a** or **4b]** and the reduction product hexahydro-2-phenanthroyl-CoA **[6]** produced by AprC (6 µm). Spectra of synthesized 2-phenanthroyl-CoA **[3]**, 9,10-dihydro-2-phenanthroyl-CoA **[4c]**, and free CoA are shown for comparison.

The reduction product was also confirmed by UV-vis analysis, which showed a different spectrum than the spectra of the enzymatically accumulated substrate dihydro-2-phenanthroyl-CoA **[4a** or **4b]** and the chemically synthesized 9,10-dihydro-2-phenanthroyl-CoA **[4c]**, and the controls, 2-phenanthroyl-CoA **[3]** and coenzyme A (Fig. 5).

**Fig 6:**
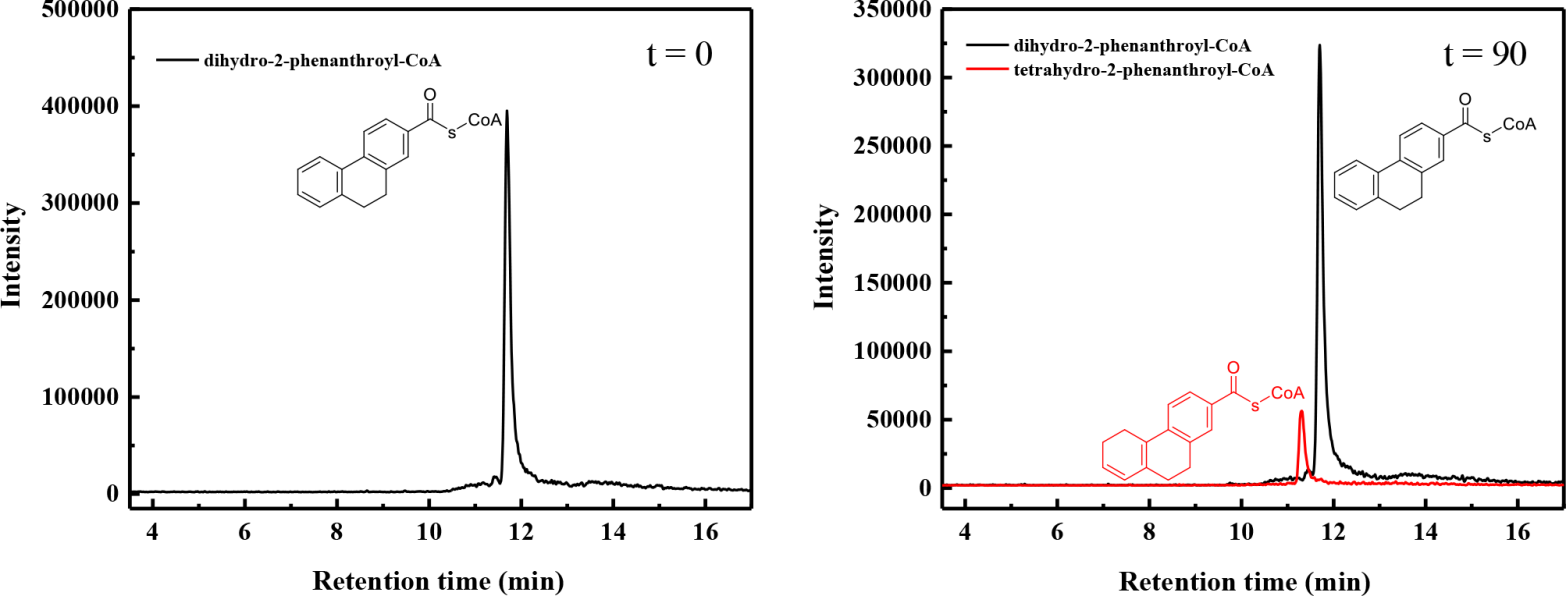
LC-MS chromatograms showing the time-dependent *in vitro* conversion of the chemically synthesized substrate 9,10-dihydro-2-phenanthroyl-CoA **[4c]** to the reduced product tetrahydro-2-phenanthroyl-CoA **[5]** by the dihydro-2-phenanthroyl-CoA reductase AprC. The black line indicates the chemically synthesized substrate 9,10-dihydro-2-phenanthroyl-CoA **[4c]** (*m*/*z* = 974, positive ion mode). The red line indicates the reduced product, tetrahydro-2-phenanthroyl-CoA **[5]**, produced after 90 min (*m*/*z* = 976, positive ion mode). The position of the saturated bond in ring III of the product tetrahydro-phenanthroyl-CoA **[5]** is only shown exemplarily because the exact structure is not known so far (Fig. S2).

We also chemically synthesized the compound 9,10-dihydro-2-phenanthroyl-CoA **[4c]** because this is the thermodynamically most stable isomer of dihydro-2-phenanthroyl-CoA **[4]**. The chemically synthesized 9,10-dihydro-2-phenanthroyl-CoA **[4c]** was converted by AprC in only one two-electron step to tetrahydro-2-phenanthroyl-CoA **[5]** with a specific activity of 3.6 ± 0.7 nmol min^−1^ mg^−1^ (Fig. 6). The reduction reaction also needed dithionite-reduced methyl viologen as electron donor, and 1 mM NADH, 50 µM FMN, and 1 mM FAD as additives to improve the reaction rate. Hence, the chemically synthesized 9,10-dihydro-2-phenanthroyl-CoA **[4c]** is most likely not the natural substrate of dihydro-2-phenanthroyl-CoA reductase enzyme because the specific activity is four times lower compared to the enzymatically produced dihydro-2-phenanthroyl-CoA **[4a** or **4b]** and can only be reduced in one two-electron step. These findings support our previously published results that the initial reduction of 2-phenanthroyl-CoA **[3]** is most likely taking place at ring 3 (23).

In anaerobic naphthalene degradation, the first two reduction steps are catalyzed by two ATP-independent type III aryl-CoA reductases reducing only ring two of the two aromatic rings. The third two-electron reduction step, which reduces the remaining benzene ring of 5,6,7,8-tetrahydro-2-naphthoyl-CoA **[11]** to hexahydro-2-naphthoyl-CoA **[12],** is catalyzed by an ATP-dependent class I aryl-CoA reductase (Fig. S3) (20). This could be attributed to the thermodynamic limitation of reducing a benzene ring where a large resonance energy has to be overcome. Analogously, the first two aromatic rings in anaerobic phenanthrene **[1]** degradation were reduced by the two ATP-independent class III aryl-CoA reductases 2-phenanthroyl-CoA reductase (AprB) (23) and dihydro-2-phenanthroyl-CoA reductase (AprC). Hence, it seems that the reduction of a higher conjugated aromatic system such as the first ring reduction in anaerobic naphthalene **[7]** degradation and the reduction of the first two rings in anaerobic phenanthrene **[1]** degradation can work without additional energy input from ATP hydrolysis. It will be interesting to study if the reduction of the remaining benzene ring of the phenanthroyl-CoA ring system can be reduced with an ATP-independent type III aryl-CoA reductase. However, this work produced first indications that the reduction of a benzene ring can also work with a type III aryl-CoA reductase of the old-yellow enzyme family. When the chemically produced 9,10-dihydro-2-phenanthroyl-CoA **[4c]** was used as a substrate for AprC it was reduced to a tetrahydro-2-phenanthroyl-CoA **[5a]** (Fig. S2). The chemically synthesized 9,10-dihydro-2-phenanthroyl-CoA **[4c]** has two isolated benzene rings (ring I and ring III) that are only connected through saturated sigma-bonds (Fig. S4). Hence, according to the knowledge of benzoyl-CoA and tetrahydro-2-naphthoyl-CoA reduction, the activation energy for the reduction of the two isolated benzene should be too high for an ATP-independent aryl-CoA reductase of type III. We speculate that the reason for the successful reduction of the benzene ring I in 9,10-dihydro-2-phenanthroyl-CoA **[4c]** could be that the 3D structure of the compound **[4c]** is still close to coplanar (Fig. S2 and S4). The hydrogenation at the 9,10-positions introduces only a slight bending of the central ring, which disrupts π-conjugation between the three fused rings only to a certain extent as compared to a perfectly coplanar phenanthrene structure. However, we speculate that the slight geometric distortion still allows for a partially delocalized π system between the two formal benzene rings rendering a more conjugated system, thus, lowering the activation energy for reduction compared to a pure benzene ring (44). Furthermore, the distortion of the ring system leads to non-planar sp2 carbon atoms which further favors reduction to alleviate unfavorable carbon bond angles. As a result, the molecule is in a partially “pre-activated” state, requiring less energy to add further hydrogen atoms and perform reduction. The only partial loss of strict coplanarity, thus, facilitates access of reducing equivalents to the π* orbitals and lowers the activation barrier for subsequent hydrogenation (45). Consequently, the second reduction step can proceed spontaneously with the energy provided by physiological reducing equivalents alone, without the need for ATP-driven activation. Hence, this side reaction of AprC with the chemically synthesized 9,10-dihydro-2-phenanthroyl-CoA **[4c]** indicates that the reduction of a benzene ring might be even possible without additional energy input from ATP due to an at least partially conjugated π-electron system that facilitates the reduction reaction.

### Prediction of the favorable isomer of the produced hexahydro-2-phenanthroyl-CoA

**Fig 7:**
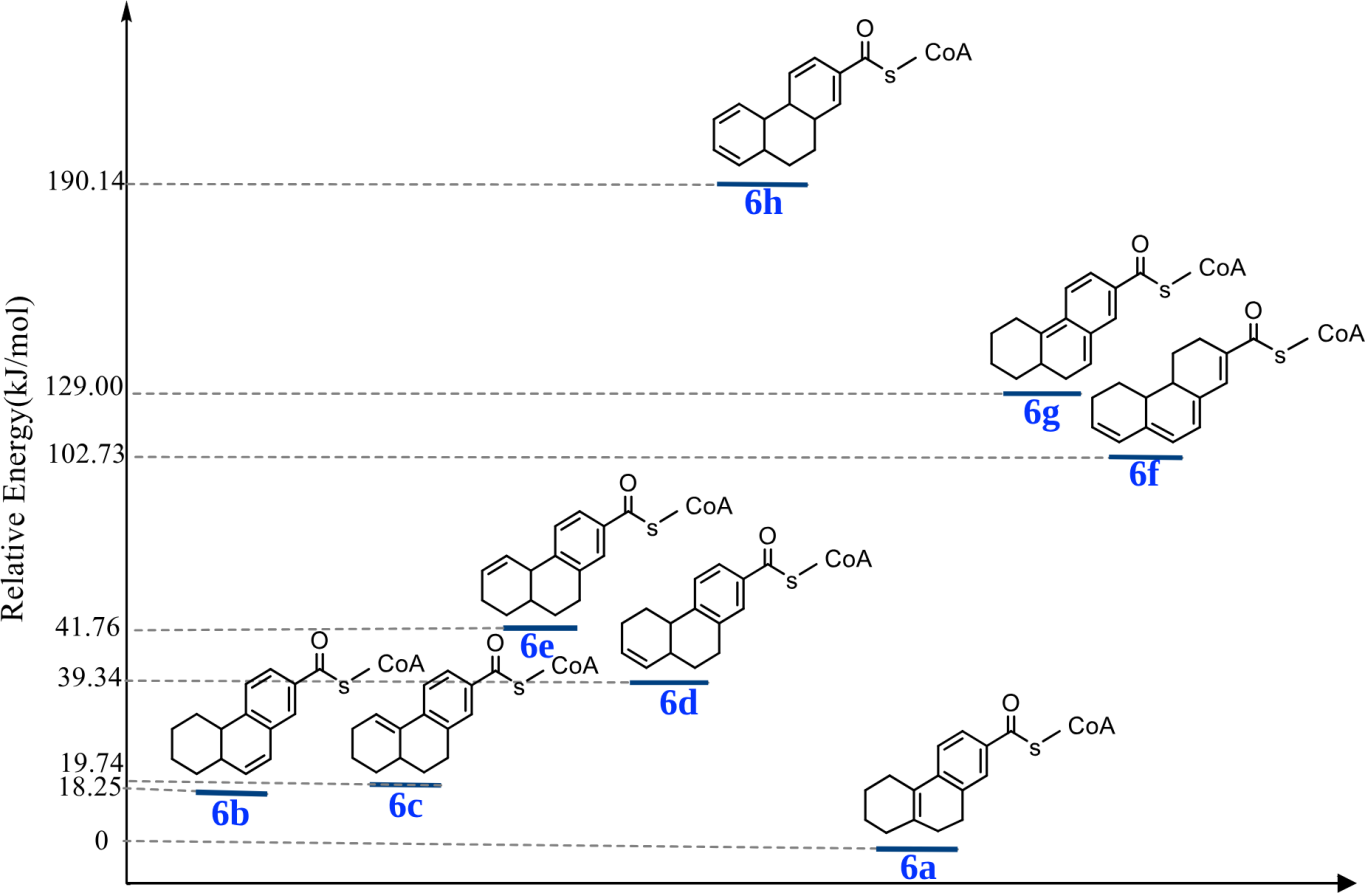
Relative electronic energies [kJ/mol] of eight possible hexahydro-2-phenanthroyl-CoA **[6]** isomers calculated at the B3LYP/6-311+G(d,p) level of theory.

Relative energy calculations (Δ*E*) of eight possible isomers of the reaction product hexahydro-2-phenanthroyl-CoA **[6]** were performed to determine possibly stable forms of the produced metabolite (Fig. 7). Isomer **[6a],** 5,6,7,8,9,10-hexahydro-2-phenanthroyl-CoA, is the energetically most stable. This preference is attributed to its optimal balance between aromaticity and conjugation. Although isomers **[6b]** and **[6c]** share similar features with isomer **[6a]**, slight differences in the double bond positions, even within the same ring, render them less stable, with noticeable energy disparities. In isomer **[6a]**, the central location of the double bond shared between the two saturated rings appears to be the most favorable arrangement due to the reduced angle strain (46) and more efficient π-overlap at the ring junction. However, the naturally produced metabolite does not necessarily have to be the chemically most stable since it has to be further reduced in the pathway, and the biochemistry will probably be optimized to produce a metabolite that can easily react in the next steps.

**Fig 8:**
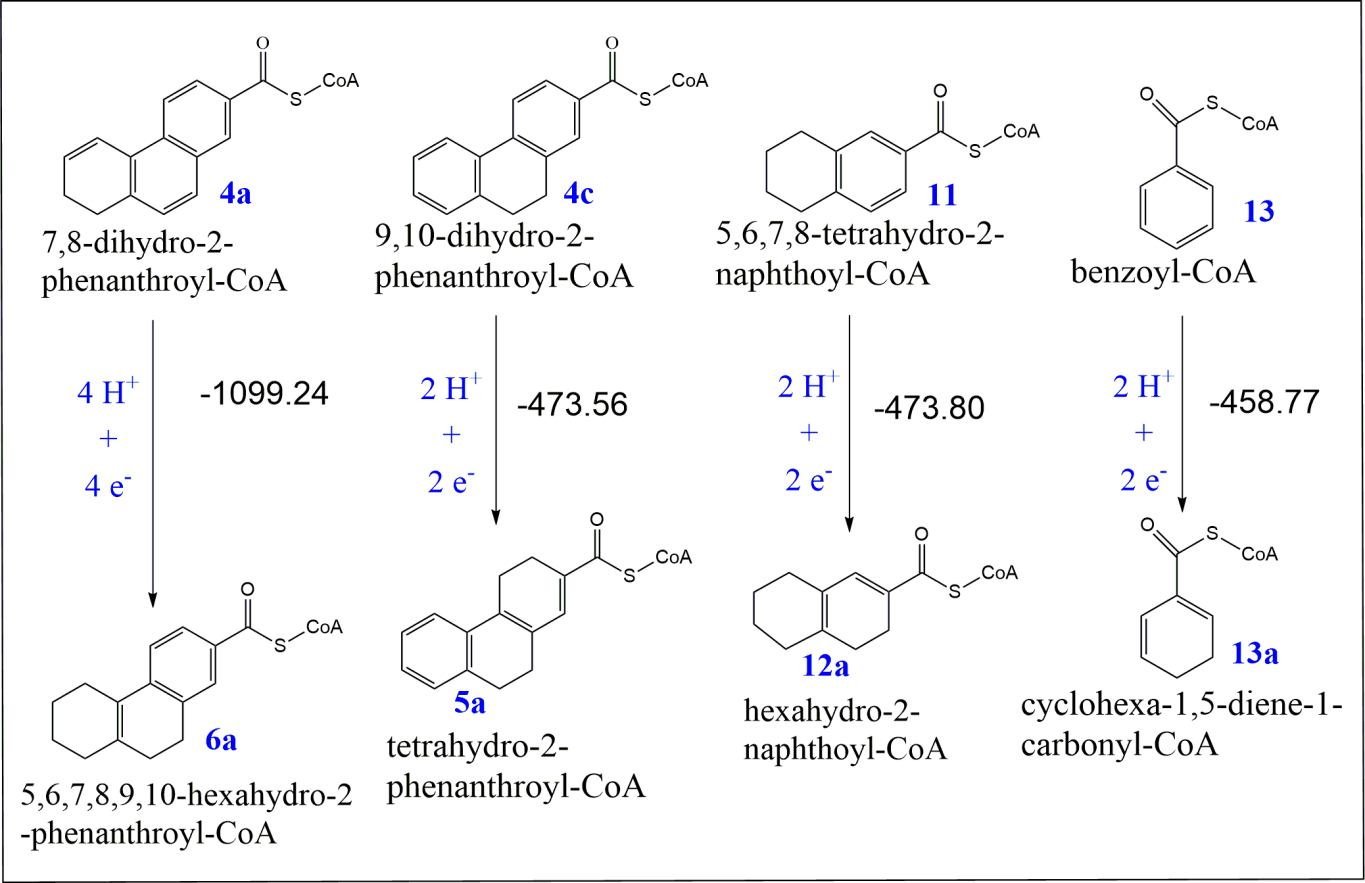
The electronic energy difference Δ*E* (kJ/mol) between substrates **[4a]**, **[4c]**, **[11]**, and **[13]** and their corresponding products **[6a]**, **[5a]**, **[12a]**, and **[13a]** was calculated at the B3LYP/6-311+G(d,p) level of theory. The electronic energy differences (Δ*E*_elec_) calculation was performed using the provided formula in the methodology section by subtracting the electronic energies of the substrates **[4a]**, **[4c]**, **[11]**, and **[13]** together with the energy of the required number of protons and electrons (provided by the electron donors such as dithionite reduced methyl viologen for the dihydro-2-phenanthroyl-CoA **[4a]**, 2-oxoglutarate as the electron donor for tetrahydro-2-naphthoyl-CoA **[11]**, and dithionite as the electron donor for benzoyl-CoA **[13]** reduction) for the reduction is subtracted from the electronic energy of the corresponding products **[6a]**, **[5a]**, **[12a]**, and **[13a]**. Since many isomer possibilities exist for the reduction products, the calculations were performed for the most stable reduced isomers.

We also calculated the electronic energies of five possible isomers of the reduction reaction of the chemically synthesized 9,10-dihydro-2-phenanthroyl-CoA **[4c]** which produced most likely a different isomer of tetrahydro-2-phenanthroyl-CoA **[5]** than the one produced from the enzymatically produced dihydro-2-phenanthroyl-CoA **[4a** or **4b]**. Isomer **[5a]** (Fig. 8; Fig. S2) was found to be the most stable compound.

The two times reductions of **[4a]** to **[6a]** released an energy difference of −1,099.24 kJ mol⁻¹. Half of this energy release (−549.62 kJ mol⁻¹) exceeded that released from the single two-electron reduction of **[4c]** to **[5a]** (−473.56), **[11]** to **[12a]** (−473.80), and **[13]** to **[13a]** (−458.77) (Table S1).

The Gibbs free energies (Δ*G*) of all the reduction reactions are negative but not equal (Table S1), indicating their spontaneity and thermodynamic favorability under constant temperature and pressure, but not to the same extent. The more negative the Δ*G* value, the more thermodynamically favorable the reduction reaction is. This is the case of the two-step reduction of dihydro-2-phenanthroyl-CoA **[4a]** to hexahydro-2-phenanthroyl-CoA **[6a]** with Δ*G*_298_**=** −174.94 kJ/mol and a released energy (Δ*H*_298_) of −245.96 kJ mol⁻¹.

The reduction of dihydro-2-phenanthroyl-CoA **[4a]** to hexahydro-2-phenanthroyl-CoA **[6a]** did not require additional energy input in the form of ATP, which is in contrast to the reduction of 5,6,7,8-tetrahydro-2-naphthoyl-CoA **[11]** to hexahydro-2-naphthoyl-CoA **[12a]** (20) or the reduction of benzoyl-CoA **[13]** to cyclohexa-1,5-diene-1-carbonyl-CoA **[13a]** (47) (Fig. 8; Fig. S3 and S5). Hence, we calculated the relative electronic energies of the reduced product isomers of tetrahydro-2-phenanthroyl-CoA **[5]**, hexahydro-2-naphthoyl-CoA **[6]**, and cyclohexa-1,5-diene-1-carbonyl-CoA **[13a]** relative to their respective starting materials (Fig. 7 and 8; Fig. S2, S3 and S5). The relative electronic energies (Δ*E*_elect_) of the most stable isomers in all three cases of **[5a], [12a]**, and **[13a]** were similar, with only a very small energy gap for the reduction of the chemically synthesized 9,10-dihydro-2-phenanthroyl-CoA **[4c]**, and the reduction of benzoyl-CoA **[13]** of approximately 0.34, and 15.03 kJ mol⁻¹ less stable, respectively. On the other hand, Δ*E*_elect_ of the most stable isomers of hexahydro-2-phenanthroyl-CoA **[6a]** was −1,099.24 kJ mol⁻¹, indicating that for one reduction step the stabilization energy was −549.62 kJ mol⁻¹ with energy gap of approximately 75.79 kJ mol⁻¹ greater stability compared with the compounds **[5a], [12a]**, and **[13a]**. This stability difference explains why the produced intermediate, tetrahydro-2-phenanthroyl-CoA, produced from the reduction of the enzymatically produced dihydro-2-phenanthroyl-CoA **[4a],** is directly further reduced to a more stable isomer of hexahydro-2-phenanthroyl-CoA **[6a]**.

According to the quantum chemical calculations (Fig. 8), the requirement for ATP is not determined by the final energy difference between substrate and product but rather by the activation energy barriers that must be overcome during the reduction process (48, 49). In ATP-dependent systems such as benzoyl-CoA **[13]** and 5,6,7,8-tetrahydro-2-naphthoyl-CoA **[11]** reduction, the breaking of aromatic stabilization involves high transition-state barriers (unstable intermediate state between reactants and products) that exceed the energy supplied by physiological reducing equivalents alone, besides the thermodynamic penalty associated with the energy loss after reduction (50). In contrast, in the phenanthroyl-CoA system, the partial delocalization of the π-electron network reduces the activation barrier sufficiently for reduction to proceed without ATP hydrolysis. Thus, the quantum chemical data explain why the final ΔE values appear small or even exergonic, while ATP dependence instead reflects differences in kinetic accessibility rather than thermodynamic feasibility.

### Homology modeling of dihydro-2-phenanthroyl-CoA reductase with other reductases

To analyze the possible reaction mechanism of dihydro-2-phenanthroyl-CoA reductase AprC, the deduced amino acid sequences of the genes coding for dihydro-2-phenanthroyl-CoA reductase (*aprC*), 2-phenanthroyl-CoA reductase (*aprB*), and 2-naphthoyl-CoA reductase (N47_G38220) were aligned with the Biotite python software package (Fig. S6) (29). The deduced amino acid sequence of AprC reductase is very similar to AprB (33.97%) and 2-naphthoyl-CoA reductase (33.09%). The alignment showed that the catalytically active Tyr197 from AprB as well as surrounding amino acids are well conserved in all three enzymes. Four Cys residues coordinating a 4Fe-4S cluster were also well conserved in all three enzymes. The hydrophobic residues coordinating FMN and FAD are less conserved but residues facilitating other interactions like hydrogen bridges, salt bridges, complexing, and ion bridges are better conserved, such as Gln 114, Arg 344, and Lys 439.

**Fig 9:**
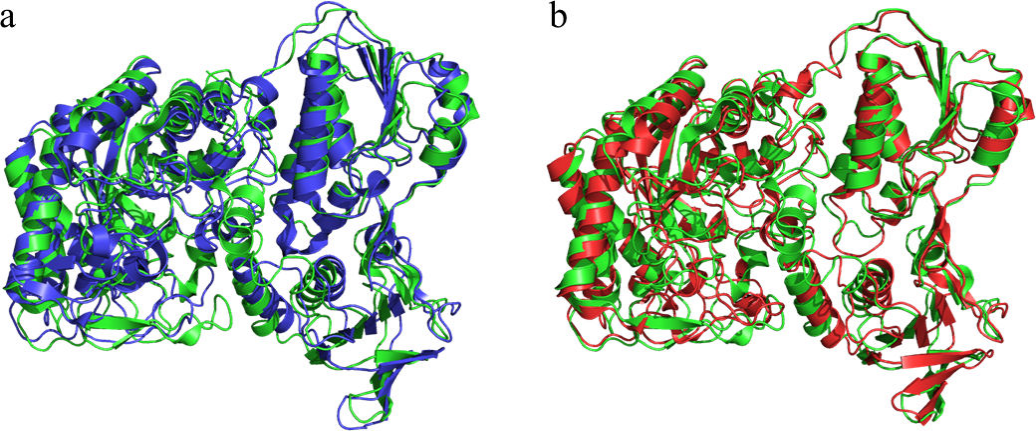
3D alignments using PyMol showing the structure of AprC (green), AprB (red), and 2-naphthoyl-CoA reductase (blue, 6QKG). (**a**) Alignment between 2-naphthoyl-CoA reductase and AprC (RMSD: 1.669 Å). (**b**) Alignment between AprB and AprC (1.834 Å).

To compare the 3D structures of 2-naphthoyl-CoA reductase, AprB, and AprC, a structure alignment in PyMol was performed (Fig. 9). The maximum rmsd (root mean square deviation) between the structures was 1.834 Å. Although the amino acid sequence of AprC aligned closely with AprB, the tertiary structure of AprC was more similar to 2-naphthoyl-CoA reductase. However, those differences are rather small indicating that the three enzymes have a very similar tertiary structure and belong to one class of enzymes.

**Fig 10:**
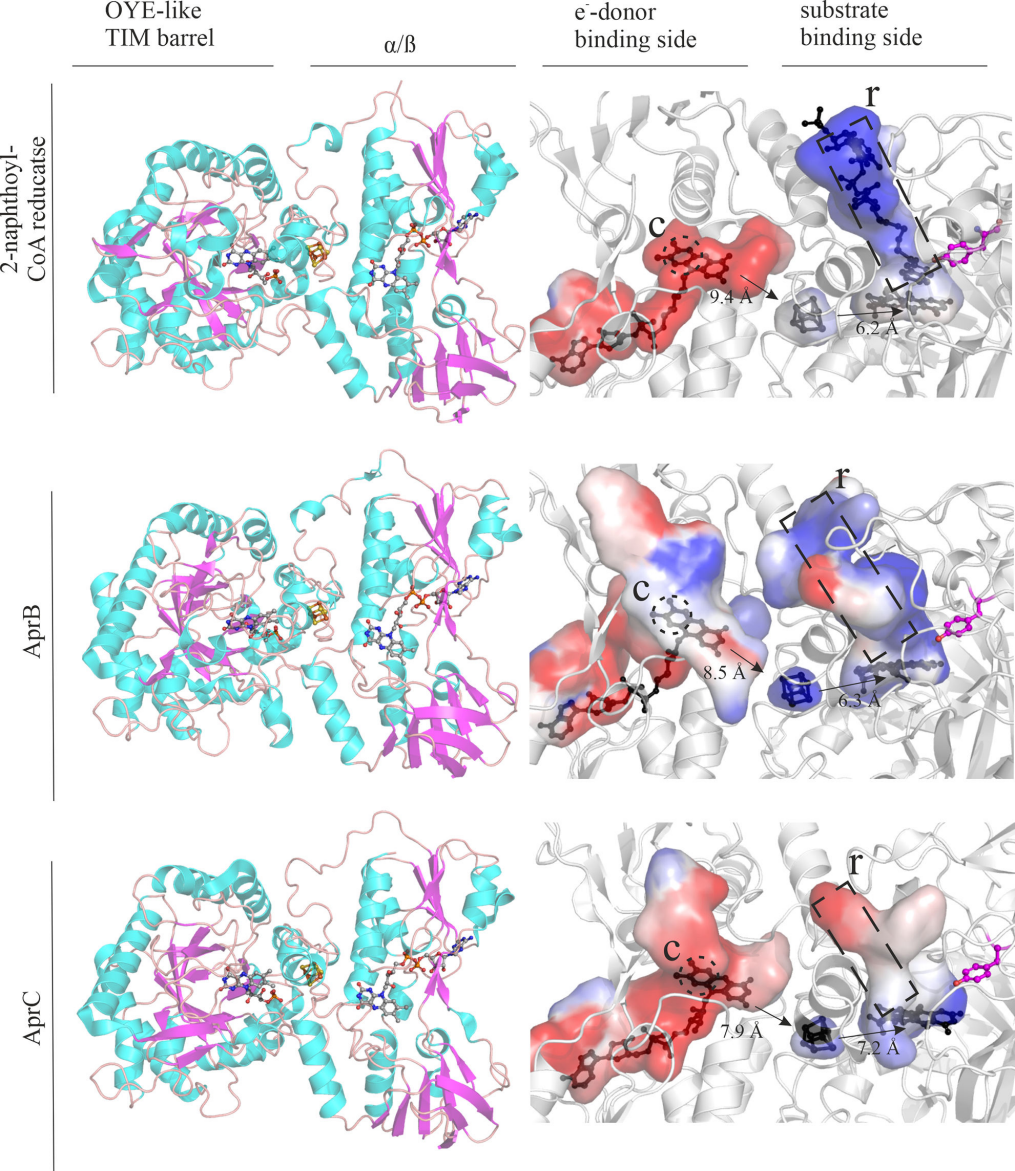
Comparison of the modeled structures of AprB and AprC with the crystal structure of 2-napthoate-CoA reductase. The figure was created with PyMol using the CavitOmiX plugin. Left column: structure comparison between the crystal structure of 2-naphthoyl-CoA reductase (NCR; PDB: 6QKG) (52), the theoretical structure of AprB, and the theoretical structure of AprC reductase. Secondary structure motifs are marked with colors (α helices: cyan, β sheets: light magenta, and loops: salmon). Right column: comparison of modeled cavities, electron transfer path, and ligand binding site of 2-naphthoyl-CoA reductase (PDB: 6QKX), AprB, and AprC. The relevant cavities are shown, and their surface is marked according to their vacuum electrostatics (negative: blue, neutral: white, and positive: red). The electron paths are marked with arrows and the theoretical distances between the cofactors [Å]. The opening of the FAD cavity leading to the outside is marked with a circle (C) and the ligand binding channel with a rectangle (R).

To compare the possible reaction mechanisms of the AprB and AprC reductases to 2-naphthoyl-CoA reductase, the protein structures were modeled with the corresponding cofactors (Fig. 10, left column). The cofactors were transplanted from existing crystal structures, of other similar enzymes that were acquired from the PDB data base, (2-naphthoyl-CoA reductase (6QKG) for AprB and 2,4-dienoyl-CoA reductase (1PS9) for AprC) into the AlphaFold structures of AprB and AprC (30, 31, 51). The secondary structures of the three enzymes matched almost perfectly; each enzyme consists of one old-yellow-enzyme-like TIM barrel domain and 2 α/β domains. To compare the possible reaction mechanisms of AprB and AprC to that of 2-naphthoyl-CoA reductase, CavitOmiX in conjunction with PyMol was used to visualize the inner cavities of the structures (Fig. 10, right column).

AprB reductase and AprC reductase showed a channel connecting the FAD cavity to the outside in the same position as 2-naphthoyl-CoA reductase. Additionally, both enzymes show negatively charged patches on the surface around the FAD cavity surface junction. This could be an indication that a ferredoxin is the unknown *in vivo* electron donor. Consequently, using NADH as the sole electron donor in the enzymatic assay retained only 40% of the enzyme’s activity in our study. This could be attributed to the role of NADH in the reduction assay to keep the flavins in a reduced state (53), and this confirms that NADH is unlikely to be the primary physiological electron donor. In the next step, after the electrons get transferred to the solvent exposed isoalloxazine moiety of FAD, they should be transferred to the 4Fe-4S cluster. The modeled distances between the FAD and 4Fe-4S cluster in AprB (8.5 Å) and AprC (7.9 Å) were in the same range as the distance between the FAD and the 4Fe-4S cluster of 2-naphthoyl-CoA reductase (9.4 Å) which allows for direct electron transfer. In analogy to the crystal structure of naphthoyl-CoA reductase, electrons are most likely transferred from the 4Fe-4S cluster to the FMN. The distances between the 4Fe-4S-clusters and FMN are similar for AprB (6.3 Å), AprC (7.2 Å), and 2-naphthoyl-CoA reductase (6.2 Å) allowing for the same electron transfer mechanism. Overall, it looks very reasonable that the electron transfer mechanism for AprC and AprB works very similarly to that of 2-naphthoyl-CoA reductase.

In 2-naphthoyl-CoA reductase, the FMN binding pocket was mostly uncharged, probably because FMN is a rather neutral compound, while the part holding the CoA moiety of the substrate was negatively charged. The binding pocket for the naphthalene ring system was partially neutral and was negatively charged near the catalytically active tyrosine residue. This is to be expected because of the hydrophobicity of naphthalene. The charge near the catalytically active residue is probably there to stabilize a putative charged intermediate. The modeled structures (Fig. 10) show that the dihydro-2-phenanthroyl-CoA reductase AprC has a very similar tertiary structure to the 2-phenanthroyl-CoA reductase AprB and 2-naphthoyl-CoA reductase. In addition, the modeling with alphafill shows that the cofactors FAD, FADH, and 4Fe4S fit in analogous spaces to AprB and 2-naphthoate-CoA reductase. The active site cavity of AprB looks similar, but the part harboring the CoA moiety of 2-phenanthroyl-CoA **[3]** has some positive and neutral charges and looks larger and less linear. The reason for this could be that the CoA part of the ligand takes a different conformation and/or is differently charged.

### Proposed reduction mechanism of dihydro-2-phenanthroyl-CoA

The high similarity of the amino acid sequence and the alpha fold structures of dihydro-2-phenanthroyl-CoA reductase (AprC), 2-phenanthroyl-CoA reductase (AprB), and 2-naphthoyl-CoA reductase indicate a similar biochemical reduction mechanism (23, 52). The inner cavities for the theoretical structures of AprB and AprC show less tightness around the binding ligands probably because they are theoretical AlphaFold structures. The high similarity of primary, secondary, and tertiary structures of the three proteins as well as the similar theoretical positions for the cofactors and the catalytic tyrosine residues indicates that the reaction mechanisms of AprB and AprC are similar to 2-naphthoyl-CoA reductase.

Evidence to date suggests that the reduction of 2-naphthoyl-CoA **[9]** (Fig. S7a) proceeds through the formation of a Meisenheimer complex-analogous intermediate **[9a** and **9b]** by 2-naphthoyl-CoA reductase, targeting the unsubstituted ring at carbon **C6**, followed by protonation at **C5** (Fig. S7a). The 2-naphthoyl-CoA reductase has to overcome the high resonance energy of the 2-naphthoyl-CoA aromatic ring at a low redox potential for flavin-mediated hydride transfer (52, 54). This is attributed to the fact that enzymes of the OYE family catalyze a hydride transfer from a reduced flavin to activated alkenes with an electron-withdrawing group, followed by the protonation of the negatively charged transition state (52, 54). The reduction of 2-phenanthroyl-CoA **[3]** to dihydro-2-phenanthroyl-CoA **[4a** or **4b]** proceeds most likely in analogy to the reduction mechanism of 2-naphthoyl-CoA **[9]** (23). According to Willistein et al. (54), 5,6-dihydro-2-naphthoyl-CoA reductase also belongs to the same OYE family, but the enzyme reduces 5,6-dihydro-2-naphthoyl-CoA **[10]** by a slightly different mechanism than the reduction of 2-naphthoyl-CoA (54) (Fig. S7b). According to Willistein et al. (54), 5,6-dihydro-2-naphthoyl-CoA reductase must have a different reduction mechanism than 2-naphthoyl-CoA **[9]** reduction because the formation of an anionic resonance structure involving the thioester carbonyl group is not possible after addition of a hydride to **C7** of 5,6-dihydro-2-naphthoyl-CoA **[10]** (Fig. S7b). In this case, the direct hydride addition turns off the resonance stability of the anionic intermediate by the CoA ester (54). Willistein et al. (54) hypothesized that an initial isomerization could enable a direct hydride transfer to **C8** of 5,6-dihydro-2-naphthoyl-CoA **[10]** (Fig. S7c), especially since enzymatic isomerization has been reported for the OYE enzyme family (55). Therefore, the reaction might start with temporary deprotonation of **C5**, followed by isomerization rearrangement to form a conjugated intermediate **[10b** and **10c]**, followed by direct hydride transfer to **C7** with resonance stabilization by the CoA ester **[10d]**, producing tetrahydro-2-naphthoyl-CoA **[11]** (Fig. S7c) (54).

**Fig 11:**
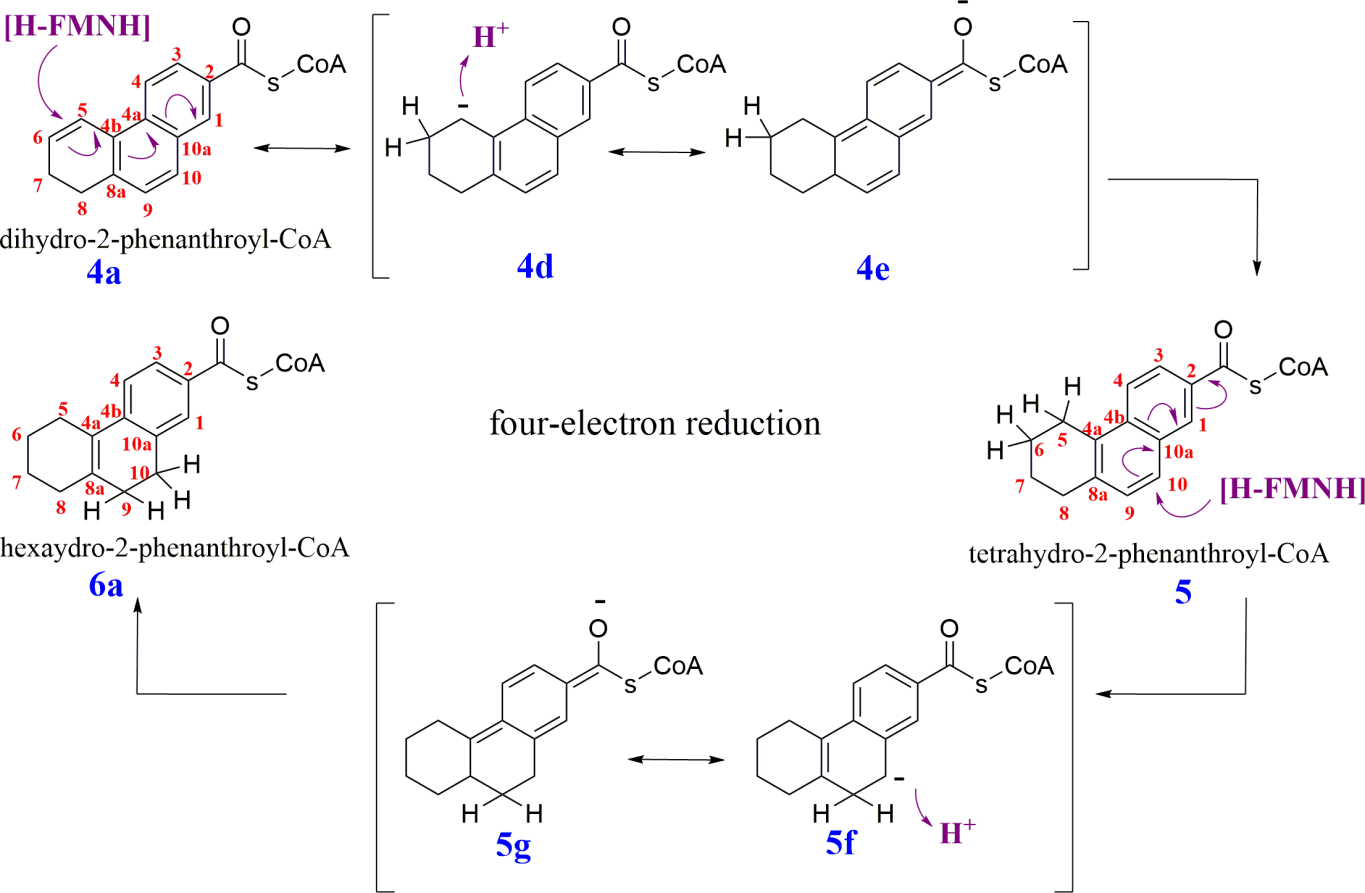
Putative two consecutive two-electron reduction steps mechanism of dihydro-2-phenanthroyl-CoA **[4a]** to hexahydro-2-phenanthroyl-CoA **[6a]** catalyzed by dihydro-2-phenanthroyl-CoA reductase AprC. Compounds **4d, 4e, 5f,** and **5g** are Meisenheimer-analogous intermediates followed by stabilized CoA-ester compounds **[5]** and **[6]**. The exact location of the reduced bonds and the site of reduction are unknown so far, and the shown structures are therefore exemplary.

In analogy to the reduction mechanism of 2-naphthoyl-CoA **[9]**, we propose a reaction mechanism for the two consecutive two-electron reductions of two double bonds by dihydro-2-phenanthroyl-CoA reductase AprC (Fig. 11). Two electrons are transferred to FMN causing a hydride transfer to **C5** of dihydro-2-phenanthroyl-CoA **[4a]** producing a Meisenheimer complex **[4d** and **4e]**. Then follows a protonation at **C5** to produce the tetrahydro-2-phenanthroyl-CoA **[5]**. The same process occurs to reduce the tetrahydro-2-phenanthroyl-CoA **[5]** to hexahydro-2-phenanthroyl-CoA **[6a]**, but the hydride is transferred to **C10 [5]** and produces another Meisenheimer complex **[5f** and **5g]**, followed by a protonation at **C10 [6a]**.

### Conclusion

The presented results indicate that at least the first three two-electron ring reduction steps in the anaerobic degradation of larger PAHs with three or more rings follow a similar strategy as shown for the smaller two-ring PAH naphthalene. The aromatic rings are reduced by type III aryl-CoA reductases belonging to the old-yellow enzyme family which only need a low potential electron donor but no additional energy input from ATP. AprC reduced the dihydro-2-phenanthroyl-CoA through two consecutive two-electron reduction steps producing hexahydro-2-phenanthroyl-CoA with unknown structure. The structure modeling of dihydro-2-phenanthroyl-CoA reductase (AprC) suggests that the enzyme probably uses the same mechanism as 2-naphthoyl-CoA reductase and 2-phenanthroyl-CoA reductase (AprB).

## Data Availability

The data supporting the findings of this study can be found in the supplemental material.

## SUPPLEMENTAL MATERIAL

Supplemental material aem.02248-25-s0001.docx Fig. S1 to S7; Table S1.

ASM does not own the copyrights to Supplemental Material that may be linked to, or accessed through, an article. The authors have granted ASM a non-exclusive, world-wide license to publish the Supplemental Material files. Please contact the corresponding author directly for reuse.
