# Supplementary material for: The ATP-independent dihydro-2-phenanthroyl-CoA reductase AprC catalyzes two consecutive two-electron reduction steps of dihydro-2-phenanthroyl-CoA to hexahydro-2-phenanthroyl-CoA in anaerobic phenanthrene degradation
Source: Appl Environ Microbiol. 2026 Mar 13;92(4):e02248-25. doi: 10.1128/aem.02248-25 (PMC13101529; doi:10.1128/aem.02248-25)
Supplement: Supplemental material — Fig. S1 to S7; Table S1. [file aem.02248-25-s0001.docx]

**The ATP-independent dihydro-2-phenanthroyl-CoA reductase AprC catalyzes two consecutive two-electron reduction steps of dihydro-2-phenanthroyl-CoA to hexahydro-2-phenanthroyl-CoA in anaerobic phenanthrene degradation**

Nadia A. Samak^1*^, Khadija Adjir^2^, Frederik Götz^1^, Alina Surmeneva^1^, Jonas Fax^3^, Gebhard Haberhauer^3^, Rainer U. Meckenstock^1*^

^1^ Environmental Microbiology and Biotechnology (EMB), Faculty of Chemistry, University of Duisburg-Essen, Universitätsstr. 5, 45141 Essen, Germany

^2^ Laboratory of Thermodynamics and Molecular Modeling, Faculty of Chemistry, University of Sciences and Technology Houari Boumediene (USTHB), BP32 El Alia, 16111 Bab Ezzouar, Algiers, Algeria

^3^ Organic Chemistry, Faculty of Chemistry, University of Duisburg-Essen, Essen, Germany

^*^ Corresponding author, E-mail:

[nadia.samak@uni-due.de](mailto:nadia.samak@uni-due.de); Tel. +49 (0)201 183-7089

[rainer.meckenstock@uni-due.de](mailto:rainer.meckenstock@uni-due.de); Tel. +49 (0)201 183-6601; Fax +49 (0)201 183-6603

**a**

ATGTCCAATCGCTTTGTGAACCTGATGTCTCCGGGCTATATCGGCAGTCTCGAAATTAAGAATCGCATCGTTACAGCGCCGCTGTGGACCGGCTATGGTGGGCGTGATGGCAGTGTTACCCCACGCACGATTGCGTACTATTCCGAAAAAGCCAAAGGTGGGTCTGGCTTGATCACCGTGGAGTATACCTACGTCGATAATATCGGCAGCAAAAGTGCCTTTGGCCAGTTGGGTATTTATGACGACGAATGCATTAAGGACTTCGCGTTACTGGCACGTGCGATTAAAGATTGGGGTGTACGTTGTGCGGTTCAATTGGCGCACGCTGGTCCGATGAAATTCCTTCCGATTTCGCCGTGGTTTGGTCCGAGCGATGGCTTTCACGACCTTAGCAATATCGGACCAATTCCGCCGGTTCCGATTACCGGTATGTCCAAAGAGGATATTTCGAACGTTATCGAGAGCTTTGCCGCAGCGGCCGAACGCGTGAAAAAGGCGGGATTTGACATGGTAGACATCCATGCTGCGCATGGCTATTTACTCACGGAATTTCTGAGTCCTCACGCCAATAAACGCACGGATGAATACGGCGGTTCACTGGAGAAACGCATGCGGTTTCCTCTCGAGGTCATCGAAGCAGTGCGCAAACGGGTTGGCCCAGATTTTCCGGTTACGGTCCGTCTTAATGGGACCGATTATGCGCCGGAAAGCCCTATTACCATCGATGAAGCACTTGTGTTTGCCTGTATGCTGGAAGAAGCAGGCGTGTCTGCCTTGCATGTGTCTGGAGGTACTGACGTTTACTTGGACAAACTCGCGACGACAACGTACGTGCGTCATGGGTTCAACGTGTATCTTGCTGAAGCCGTCAAGAAAAAGGTCAAAATTCCGGTTATTGCGACCGGCGGCATCACAACTCCGGCTTTCGCTGAGGAGATTCTGGAGGAAGGCAAAGCTGATTTCATCGCCTTAGGCCGGCCAGTTCTGGCCGATCCGTGTTGGGTGCGCAAGATTGAGGAAGATCGTCCGGAGGATATTGTGCCATGCATCCGCTGCAACGATGGCTGCGTTCGTCGCACAAGCGGGTTCTTTCACGCGACCTCGTGTGCCGTAAATCCGCGTATGGGGTTTGAAGGCATTCGCACCATTGCACCGCTGATCAAACGCAAAAATATCGCCATTATTGGTGGTGGGCCAGGTGGGATGGAGGCTGCACGTCTTGCGAAGTTACGCGGCCATGATGTAACCCTGTATGAGAAACGTGAATTGGGCGGCGCACTCATCGAAGCGTGTTGGGACCAGGAACTGAAACGTGATATCCCGCTGTTGCTCAATTACTATCGCACGCAGATGAAAAAACTGGATATCAAAATCGTCAAAGAGGAAGCGTCGATTGGGACGGTAGTGCGCGGTTGCTTTGACGCGGTTATTGTGGCGAATGGAGCAGTACCTCAGAAACTGGATATTCCGGGAACCGATAAACCTCATGTGTATCAAGCACTGGACGTCACTCGCGGTCGTGACAAAGAACTGGGTAATATCGTCATTATCATCGGTGGAGGCGTGCTGGCCTGCGAAATTGCACTGTCGCAAGTGCGCAAAGGGAAACGCGTGATCATGACTGCGCCAGAAGGGTGCTATGCGGGAGAATACGAGATTGGCGGTGATAACATCCCGAATCGCATTGCCCTGATGGAGGAATTACGGAAAAACAACGTGGAGATCAACTTGTGCCTGTTACCGAAAGAAATTACCGACCAAGGCATTATCTCACTGGATAAGGACGGTAAGGATCGGATCTTCAAAGGCGATTCAGTGATTATTTGCCGTGGCTTCTTACCGGATCGTAAGCTGACCAACGAGCTGCGTCAGAAACTGAAAGATGTCTATACCATTGGCGATTGTGTGGAAGCACGTTTCATCTATGATGCCATCCACGAAGGTTGGTTAGCTGGTAA


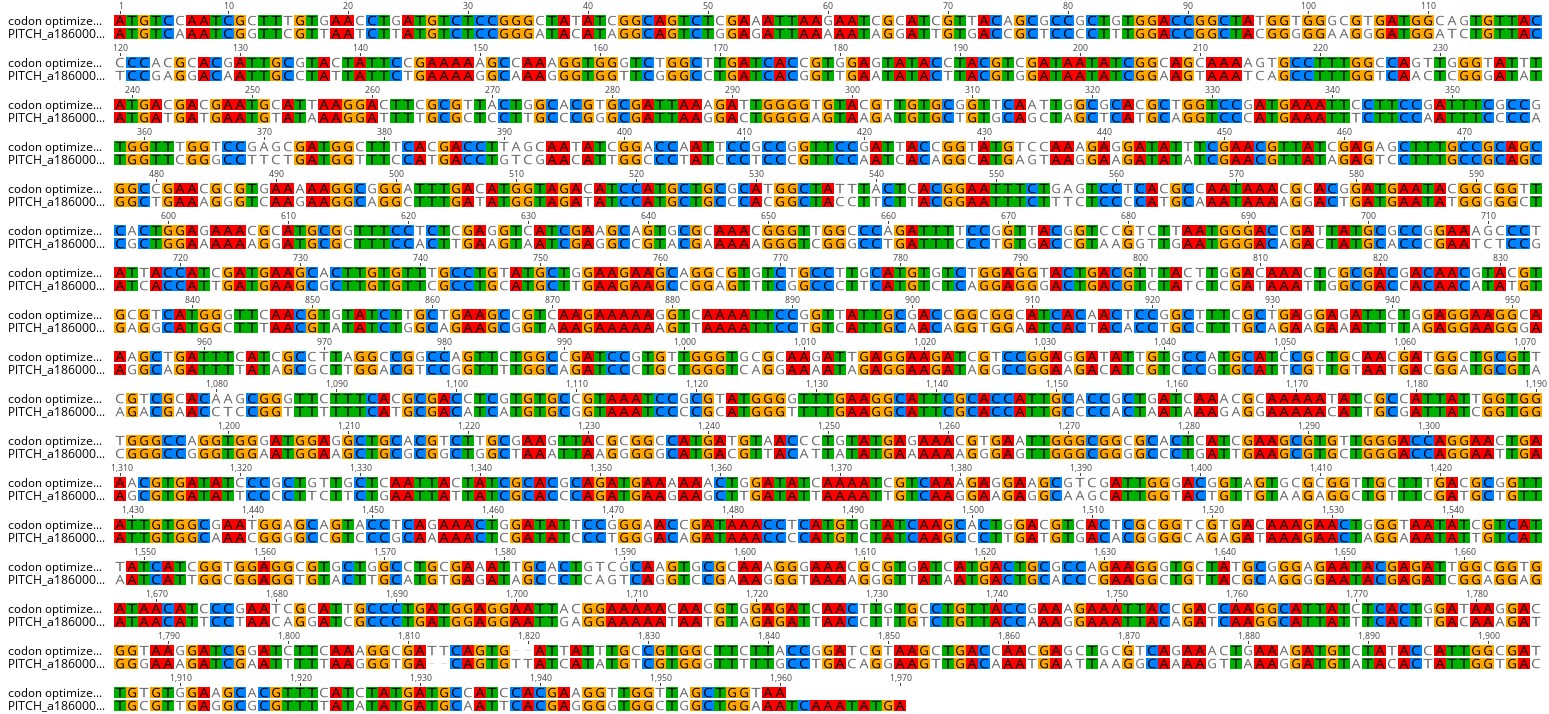


**b**

**Fig. S1. (a)** codon optimized gene sequence of PITCH_a1860005 (*aprC*) (1958 bp). **(b)** Sequence alignment of the native and codon optimized *aprC* gene.


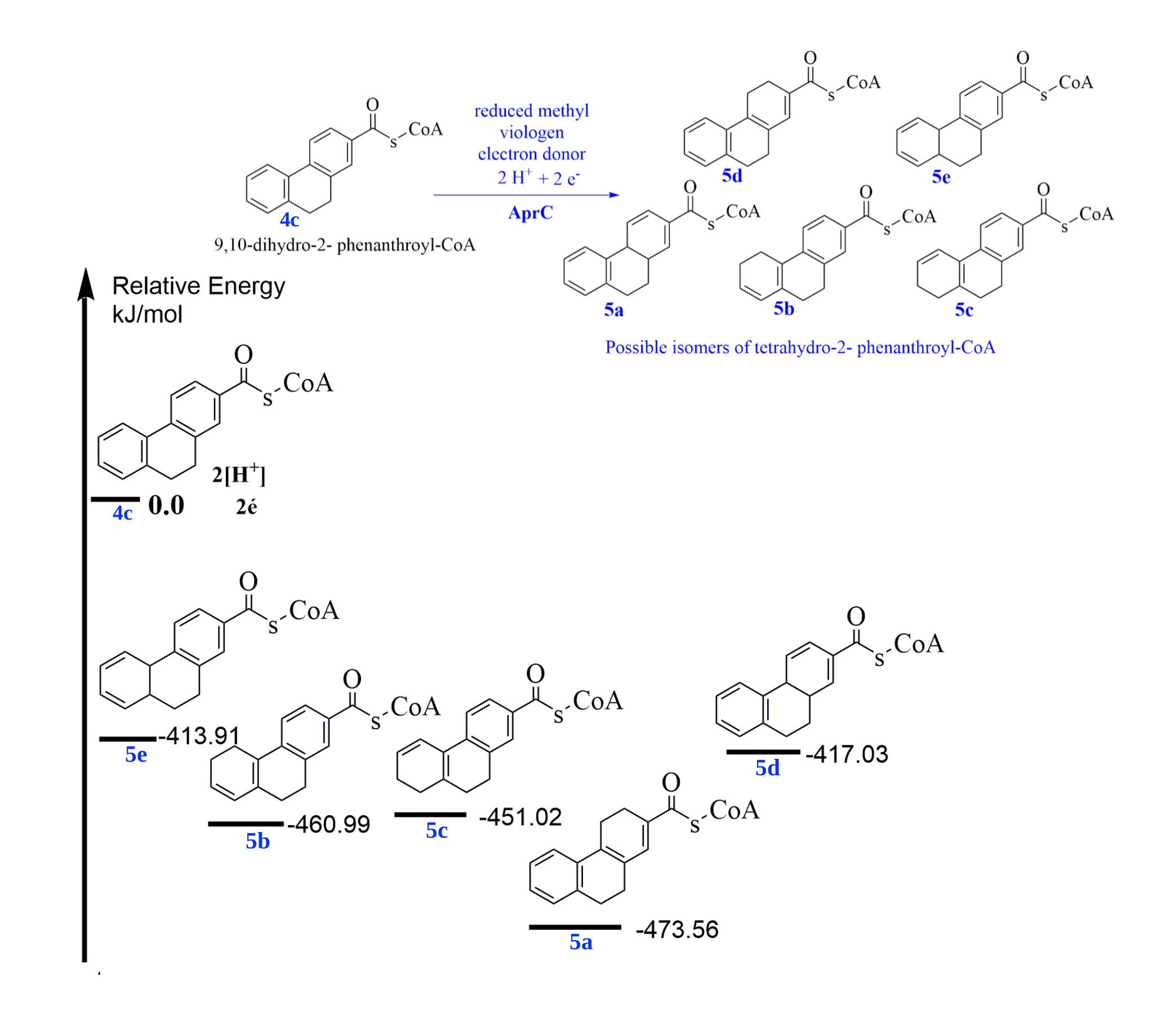


**Fig. S2.** Relative energies (in kJ/mol) of five possible tetrahydro-2-phenanthroyl-CoA **[5]** isomers, produced from the reduction reaction of the chemically synthesized 9,10-dihydro-2-phenanthroyl-CoA **[4c]**, calculated at the B3LYP/6-311+G(d,p) level of theory.


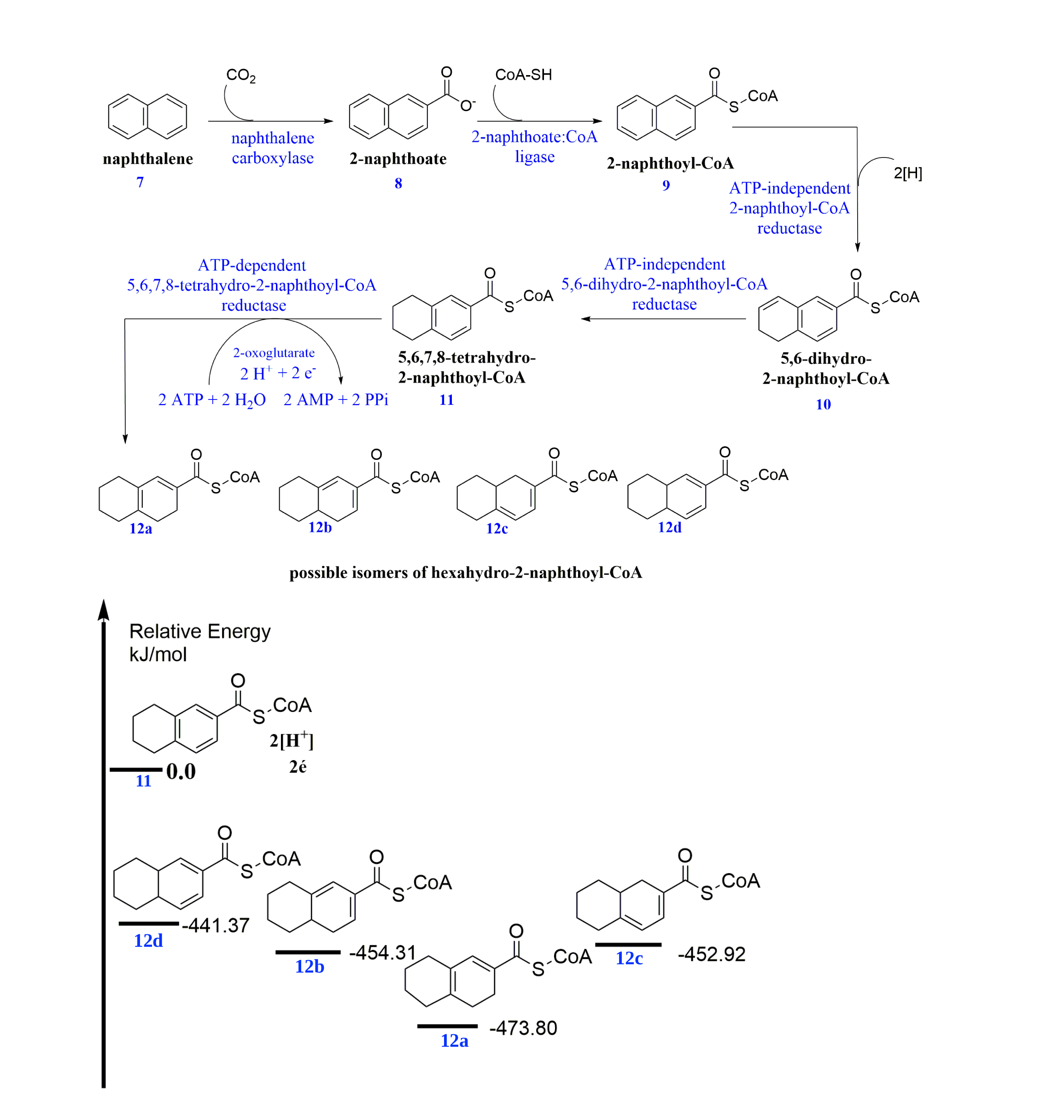


**Fig. S3.** Upper image indicates the pathway of anaerobic naphthalene **[7]** degradation. Naphthalene **[7]** is activated by naphthalene carboxylase to 2-naphthoate **[8]** which is then converted to 2-naphthoyl-CoA **[9]** by 2-naphthoate:CoA ligase. 2-Naphthoyl-CoA **[9]** follows stepwise two electron reduction catalyzed by an ATP-independent 2-naphthoyl-CoA reductase to produce 5,6-dihydro-2-naphthoyl-CoA **[10]**, which undergo another two-electron reduction catalyzed by an ATP-independent 5,6-dihydro-2-naphthoyl-CoA reductase to produce 5,6,7,8-tetrahydro-2-naphthoyl-CoA **[11]**. The latter is further reduced to hexahydro-2-naphthoyl-CoA **[12]** by an ATP-dependent 5,6,7,8-tetrahydro-2-naphthoyl-CoA reductase enzyme.


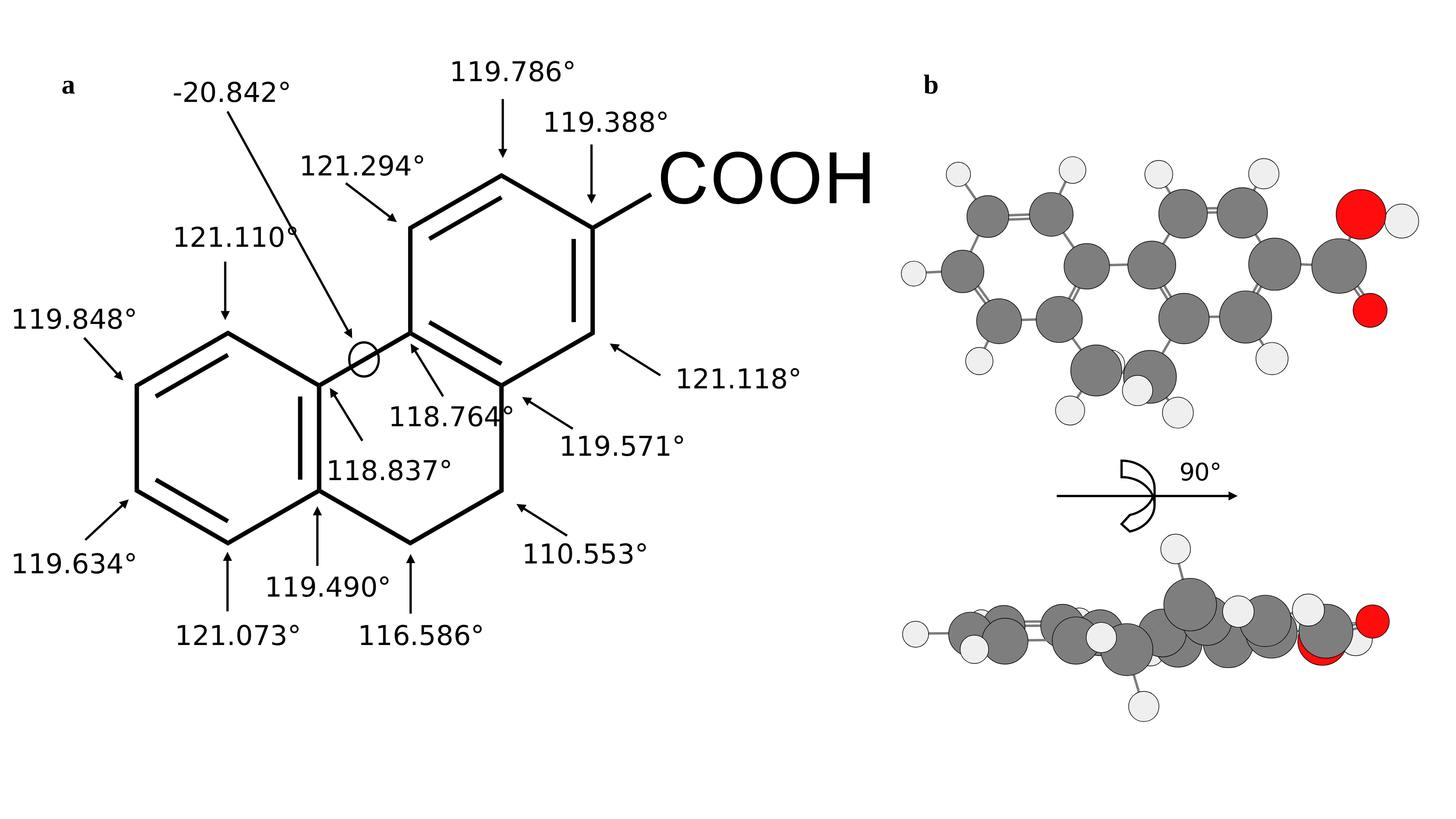


**Fig. S4.** 3D structure of 9,10-dihydro-2-phenathroic acid (the free acid of compound **[4c]**) showing that the compound is not fully coplanar. The lowest energy conformation was found with Orca using the GOAT (1). The structure was further energy minimized using a B3LYP functional, D3BJ Becke-Jones dampening, and a def2-SVP base set (2-4). The C-C-C angles of the aromatic rings and the 2 sp3 carbons on the second ring are indicated (**a)**. The angle (-20.842°) that is marked with the circle is the dihedral angle between the two aromatic rings. The compound is somehow similar to the biphenyl which is planar in the solid phase with an angle of -42.286°. Simulated 3D structure of 9,10-dihydro-2-phenathroic acid from two angles confirming that the compound is non-planar and not fully coplanar (**b**).

**Table S1.** Electronic energy differences (ΔE_Elect_), enthalpy changes (ΔH_298_), and Gibbs free energies (ΔG_298_) between the substrates **[4a]**, **[4c]**, **[11]**, and **[13]** and their corresponding products **[6a]**, **[5a]**, **[12a]**, and **[13a]**, respectively, calculated at the B3LYP/6-311+G(d,p) level of theory.

| **Substrate/corresponding product** | **ΔE_Elect_**  (kJ /mol) | **ΔH_298_** (kJ /mol) | **ΔG_298_**  (kJ /mol) |
| --- | --- | --- | --- |
| **[4c] / [5a]** | -473.56 | -71.93 | -36.13 |
| **[11] / [12a]** | -473.80 | -72.19 | -38.51 |
| **[13] /** **[13a]** | -458.77 | -60.33 | -25.67 |
| **[4a] /** **[6a]** | -1099.24 | -245.96 | -174.94 |

**
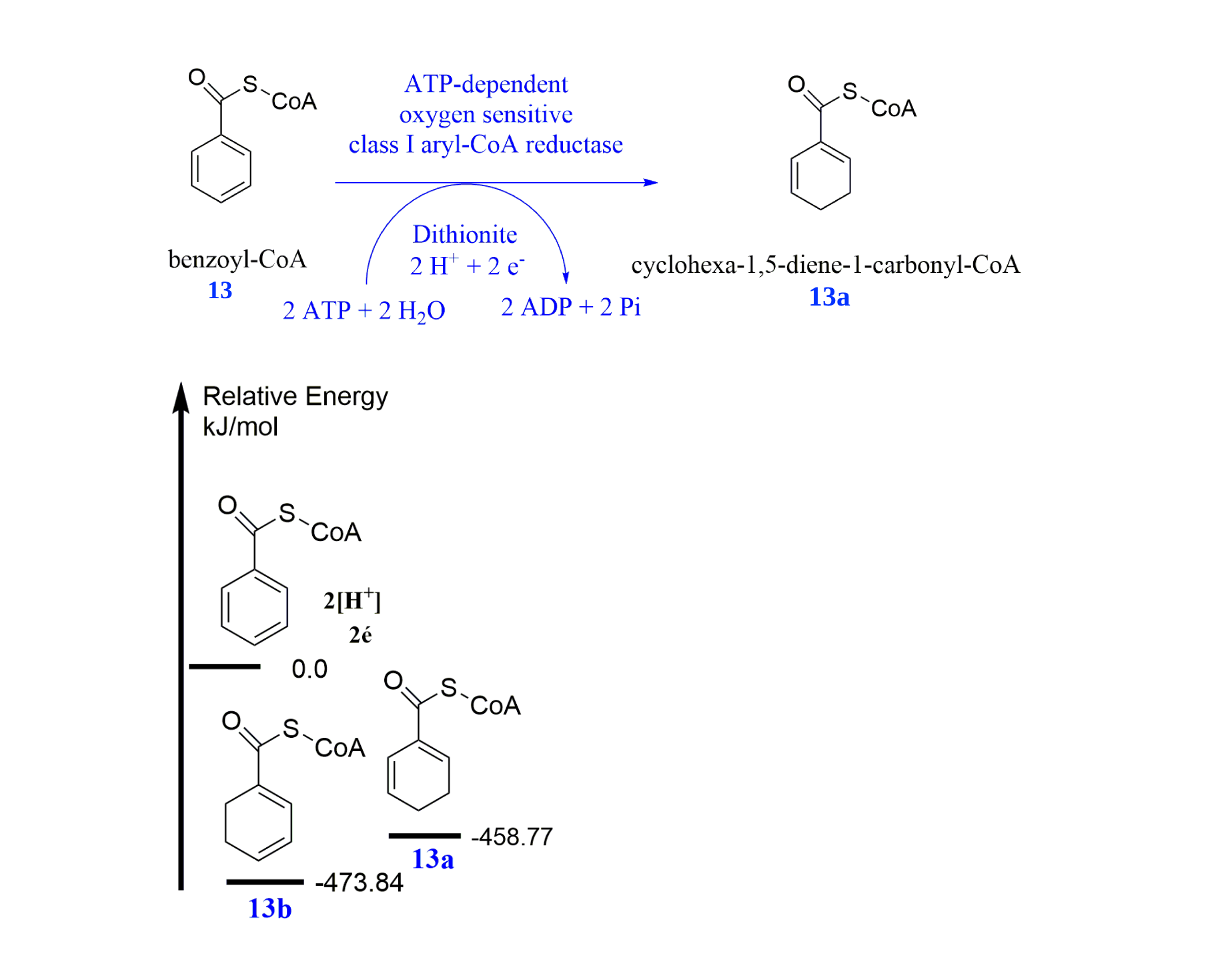
**

**Fig. S5.** Relative energy (in kJ/mol) of cyclohexa-1,5-diene-1-carbonyl-CoA **[13 a or 13b]**, produced from the reduction reaction of benzoyl-CoA **[13]**, calculated at the B3LYP/6-311+G(d,p) level of theory. The upper equation was modified from (5).


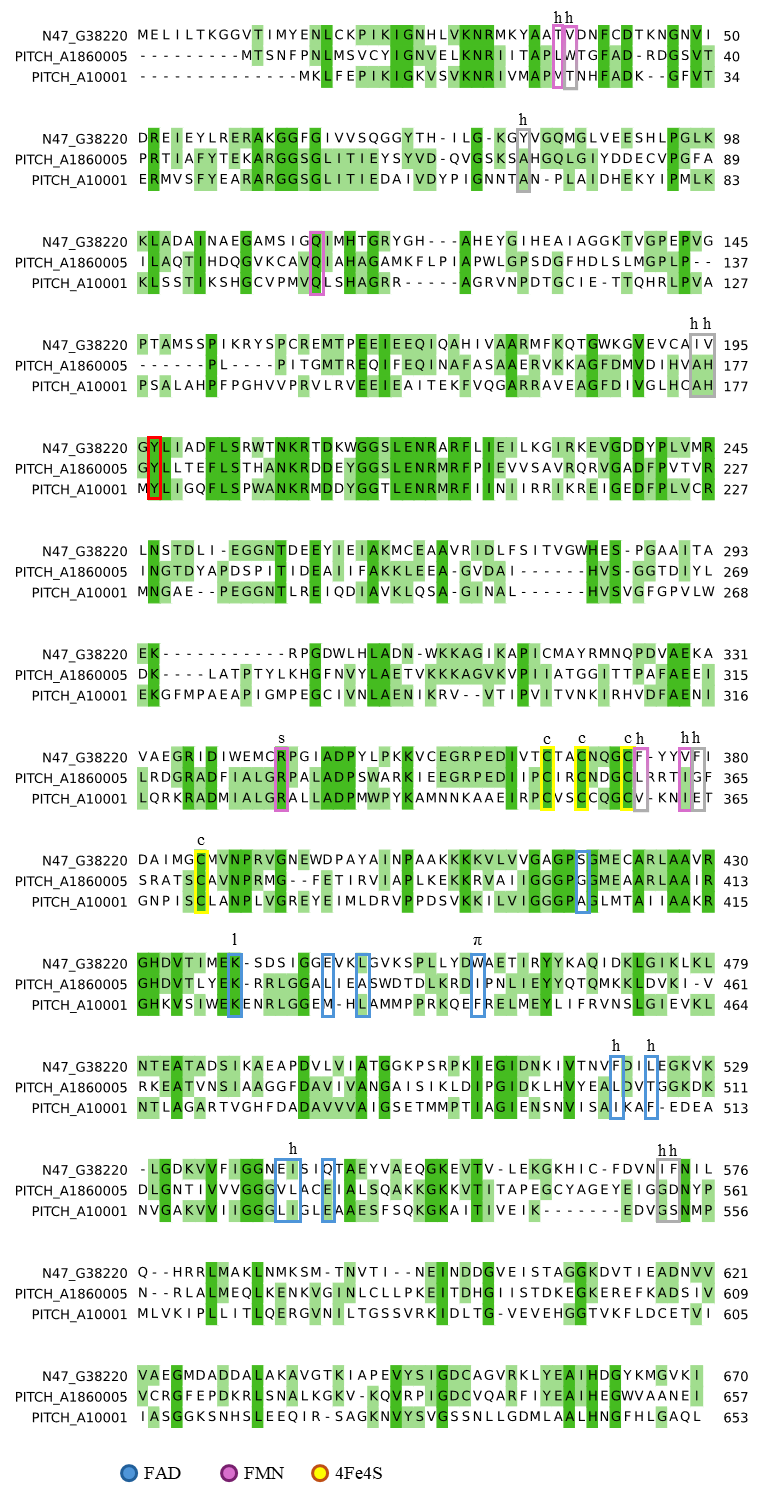


**Fig. S6.** Sequence alignment of 2-naphthoyl-CoA reductase (N47_G38220), dihydro- 2-phenanthroyl-CoA reductase (PITCH_A1860005, aprC)), and 2-phenanthroyl-CoA reductase (PITCH_A10001, aprB). The similarity between the amino acids is marked in a colour gradient from white to green. Amino acids that interact through their side chains with FAD in 2-naphthoyl-CoA reductase were marked in blue, with FMN in pink, and with the iron sulphur cluster in yellow. The types of interactions were marked as following: no mark=hydrogen bridge, h=hydrophobic interaction, s=salt bridge, c=complex, I=ion-bridge. The conserved catalytically active Y was marked in red.

The lower image indicates the relative energies (in kJ/mol) of four possible hexahydro-2-naphthoyl-CoA **[12]** isomers calculated at the B3LYP/6-311+G(d,p) level of theory. The upper image of the naphthalene degradation mechanism was modified from (6).





**Fig. S7. (a)** Reduction reaction mechanism of 2-naphthoyl-CoA **[9]** using 2-naphthoyl-CoA reductase enzyme proceeds via hydride transfer from a reduced flavin to carbon **C6**, followed by protonation at **C5** to stabilize the CoA ester. **(b)** Impossible reduction mechanism of 5,6-dihydro-2-naphthoyl-CoA **[10]** via direct hydride transfer to **C7** due to the resonance instability of the CoA ester anionic intermediate. **(c)** Possible reduction mechanism of 5,6-dihydro-2-naphthoyl-CoA **[10]** using 5,6-dihydro-2-naphthoyl-CoA reductase enzyme proceeds via isomerization starting by temporary deprotonation at **C5** to transfer a hydride to **C7** to stabilize the CoA ester and produce the tetrahydro-2-naphthoyl-CoA **[11]**. Figure adapted from (7).

**References**

1. Neese F. 2025. Software update: The ORCA program system—version 6.0. Wiley Interdiscip Rev: Comput Mol Sci 15:e70019.

2. Tirado-Rives J, Jorgensen WL. 2008. Performance of B3LYP density functional methods for a large set of organic molecules. J chem theory comput 4:297-306.

3. Behara PK, Jang H, Horton JT, Gokey T, Dotson DL, Boothroyd S, Bayly CI, Cole DJ, Wang L-P, Mobley DL. 2024. Benchmarking quantum mechanical levels of theory for valence parametrization in force fields. J Phys Chem B 128:7888-7902.

4. Grimme S, Ehrlich S, Goerigk L. 2011. Effect of the damping function in dispersion corrected density functional theory. J comput chem 32:1456-1465.

5. Möbitz H, Boll M. 2002. A Birch-like mechanism in enzymatic benzoyl-CoA reduction: a kinetic study of substrate analogues combined with an ab initio model. Biochem 41:1752-1758.

6. Weyrauch P, Heker I, Zaytsev AV, von Hagen CA, Arnold ME, Golding BT, Meckenstock RU. 2020. The 5,6,7,8-tetrahydro-2-naphthoyl-coenzyme A reductase reaction in the anaerobic degradation of naphthalene and identification of downstream metabolites. Appl Environ Microbiol 86:e00996-20.

7. Willistein M, Haas J, Fuchs J, Estelmann S, Ferlaino S, Müller M, Lüdeke S, Boll M. 2018. Enantioselective enzymatic naphthoyl ring reduction. Chem Eur J 24:12505-12508.
